# Investigating consumers’ experiences with community supported agriculture: Convergent parallel design methods

**DOI:** 10.1371/journal.pone.0303184

**Published:** 2024-05-13

**Authors:** Yuanyuan Huang, Yuhan Wang, Younghwan Pan

**Affiliations:** Department of Smart Experience Design, Kookmin University, Seoul, Korea; Shandong University of Science and Technology, CHINA

## Abstract

Community-supported agriculture (CSA) represents a collaborative model where local farms and community members form partnerships to facilitate the direct delivery of fresh produce from farms to consumers. This study primarily investigates the experiences of current CSA members, focusing on the key factors influencing their retention intentions. Employing a convergent parallel mixed methods approach, this study gathers and analyzes both quantitative data (such as factors affecting members’ retention intentions) and qualitative data (derived from interviews reflecting members’ perceptions of their CSA experiences). The integration of these datasets provides a comprehensive understanding of the factors that shape CSA membership dynamics. The research findings underscore that *Convenience*, *Product Quality*, and *Positive Interactions* are pivotal factors that contribute to members’ *Intentions* to continue their CSA memberships. These insights are crucial for enhancing the services provided to CSA members and hold significant implications for the broader scope of CSA membership research. This study not only fills a critical gap in understanding the Chinese CSA context but also contributes to the global discourse on sustainable agriculture practices and community engagement.

## 1. Introduction

Community Supported Agriculture (CSA) constitutes a sales model where local farms and community members collaborate to deliver farm-fresh produce directly to consumers [1]. This model is part of a wider global initiative that fosters localized food systems through a partnership-based approach, wherein both consumers and producers share in the risks, responsibilities, and benefits of agricultural production [2]. Traditional CSA is characterized as a direct selling strategy, where farmers offer their products at retail prices. This approach theoretically allows CSA farmers to augment their income by eliminating intermediaries, thus directly linking the production and consumption within a community [2]. Since its inception in 1986, the number of CSAs in several countries has increased rapidly [3,4]. Early CSA "models" have diversified over time and with changing circumstances [5–8]. The pattern changes from a need for food to a need for experiences [9]. Beyond the procurement of local produce, CSA members place significant value on their direct engagement with local farms. This interaction not only cultivates a sense of ownership regarding their food sources but also enhances their sense of community belonging. This aspect distinctly differentiates CSA from other food purchasing channels by fostering a deeper, more personal connection between consumers and the source of their food [9].

The survival of the CSA depends on the support of its members, so maintaining a good relationship between farmers and members is one of the key elements of the CSA. It is important to understand the member experience and improve the quality of service, which can reduce the self-exploitation of farmers and avoid reducing the price of products to retain customers [10–14]. In previous CSA membership studies, consumer behaviors, attitudes, and beliefs have a crucial impact on their willingness to join and remain loyal [15–17]. Demographic characteristics and lifestyles of consumers are used to predict the likelihood of future CSA membership [18]. These findings are used to improve marketing strategies, such as word of mouth and advertising media, which can attract more new members to CSA [18]. Research on membership retention has focused on surveys of CSA member satisfaction [19–22]. Prior research has examined factors such as price, convenience, trust, local food, product quality, customization, and online engagement that have been shown to positively influence membership joining and maintaining loyalty [3,21,23–30].

The advent of information technology has significantly transformed Community Supported Agriculture (CSA), particularly in the realms of membership management and farmer-member communication [31,32]. Studies indicate that leveraging the Internet and social media platforms enhances interaction and strengthens relationships between farmers and CSA members [32]. While these digital tools positively influence the CSA experience, they do not supplant the tangible experience of collaborative food cultivation on farms. CSA operators are increasingly exploring novel market entries, such as e-commerce, to expand product sales and forge collaborative ventures, thereby sustaining farms, attracting new consumers, and maintaining member loyalty [9]. Integration of e-commerce in CSA, emphasize the dissemination of CSA concepts and member engagement through digital content management, including instructional and recipe sharing, coupled with feedback mechanisms. Some members are willing to pay for this content, which creates an additional income for the farm. This strategy has beneficial in the CSA model [9]. Furthermore, the use of social media commerce by sellers to provide services to buyers is becoming increasingly commonplace [33].

While extensive research has been conducted on factors influencing CSA consumer engagement and membership renewal, a deep examination of the CSA membership experience remains limited. This gap is largely attributed to recent developments in logistics that have enabled fresh agricultural products to be delivered more quickly, facilitating a transition from traditional offline to online sales models in CSA. Previous studies focused on member satisfaction within the traditional CSA framework, examining how product diversity and risk preferences impact member experiences. However, as CSA models evolve with increasing online integration, member experiences and engagement are also changing. Despite this evolution, there is a dearth of contemporary research from the perspective of member experience in this new context. Moreover, while some studies suggest positive impacts of online services on member experiences, particularly in terms of online participation, comprehensive research on the online versus offline member experience and its influence on future retention intentions is scarce. This study, therefore, aims to fill this gap by exploring the factors influencing the CSA experience from the perspective of current CSA members, particularly in the context of these emerging online models.

To fill this gap our research focused on exploring the experiences of existing CSA members, examining the factors that influenced their experiences and how these factors influenced their intention to participate again in the future. A hybrid research method of convergent parallel design is used, which combines the traditional features of quantitative and qualitative research methods. Through this research design, we were able to link the related exploration of member experience with the measurement of member retention intention. The study’s first phase involved the simultaneous collection of experience evaluation questionnaire data from 250 members and semi-structured interview data from 11 members. In the second stage, we analyzed these data separately to obtain the results of quantitative evaluation data and qualitative exploration of influencing factors. In the third stage, we comprehensively compared the two sets of results and discuss them in detail.

CSA is a sustainable food production practice that shows positive performance in terms of social, environmental, and ecological sustainability [26,34–37]. CSA provides its members with healthy foods that improve their eating patterns, health levels, and well-being [1,34,38,39]. To expand the impact of CSA and fully realize its value, consumers need to be willing to continue to join CSA. Membership retention has become a crucial part of CSA’s future development [12]. Retaining existing CSA members can reduce resource investment compared to constantly recruiting new CSA members [12]. Therefore, a deeper understanding of which CSA characteristics influence membership retention and how to better retain existing members is critical [40]. Although these researchers have conducted a variety of studies on the retention factors of CSA members, the specific aspects have not been identified. The exploration of these influencing factors helps to reveal the membership needs of the CSA that are being updated and how to adapt to new opportunities and new competition.

## 2. Literature review

### 2.1 Community-supported agriculture

Community Supported Agriculture (CSA) is a model that aims to establish direct sales between farmers and consumers (especially farm members or shareholders) [41]. This model requires consumers to reach long-term agreements with producers [2]. Consumers typically pay farmers a fixed price prior to the growing season, and members will receive crops from the farm on a regular basis and share risk of crop production with the farm [1,42]. As one of the alternative agricultural system models, the core value of CSA is to promote localized food systems and emphasize the importance of creating sustainable food systems that benefit both farmers and consumers [2,43].

CSA is a community that allows members to share common goals, and participating members finance the farm by purchasing shares before the planting season so that a portion of the food is available during the harvest season [8]. By sharing the production risk with the farm, this financing method provides a guarantee for the income of the farmers in the next year and enables the farmers to purchase the materials needed for production [8,44–46]. The cost of joining the CSA is now not limited to the purchase of a one-year share, and in countries such as Turkey, they have adopted a "pay as you go" payment strategy to attract more members to join [47,48]. In the United States, all-share purchases are available for two to five people, and members also have the option to purchase half shares for one to three people to meet the needs of different members [49]. CO-CSA provides subsidies or offset costs for some low-income families [50]. CSA members in China can pay a lower upfront payment and make regular payments upon receipt of the product [37,51]. Most CSA farmers in China are not traditional farmers with rich experience, but younger generations of new farmers. Although they do not have much actual agricultural experience, most of them have higher education a more innovative spirit, and environmental awareness [51]. Unlike traditional eco-farming, these new farmers operate CSA farms more as small companies [52]. Nearly half of the farms have embraced online sales, utilizing their mini-programs or leveraging Taobao—a prominent online shopping platform in China, known for its wide reach and versatility in product offerings. These farms also collaborate with farmers’ markets to establish convenient pick-up points, or they use express delivery services to distribute products directly to members. This integration of online platforms like Taobao demonstrates a significant shift in how CSA farms are adapting to digital marketplaces to expand their reach and convenience [51]. They use new channels and models such as the Internet, social media, and farmers markets to promote their organic food and flexibly adjust their food production varieties [52].

Early CSA "models" have diversified over time and with changing circumstances [5–8]. Managers need to realize the best development path considering specific realistic conditions based on the basic characteristics of CSA [53]. Currently, CSA is beginning to actively respond to consumer preferences, for example, more pick-up sites, wider newsletters, and online blogs, and responding to consumer preferences becomes a mechanism [54]. The focus of the CSA has shifted from farmers to consumers, where the original CSA emphasized how to serve farmers, and now it seems that farmers are thinking about how to better serve their customers [9]. Farm operators are constantly looking for new ways to enter non-traditional markets, including joining e-commerce, increasing sales of value-added and processed products, and obtaining outside resources for cooperation, which can be used to maintain the farm, attract new consumers, and retain shareholders [9]. In addition, more and more e-commerce applications are being developed to better meet the needs of CSA shareholders for online ordering, supplementary purchases, product information, food matching, etc., which are also seen as part of a larger customer relationship management program [9].

### 2.2 Community engagement

In a broad sense, "communities" are formed based on location, common interests, advocacy, or profession [55,56]. Community definitions, needs, and motivations lay the foundation for community engagement [57]. Researchers argue that engagement in civic agriculture can foster social interaction and promote the development of democratic communities [58,59], especially in CSA contexts where communities are built around issues such as food, land, and the environment [13]. As an embodiment of citizen agriculture [60], CSA has an important mission to promote the development of democratic communities [58,59]. CSA is a social innovation with a community of producers and consumers at its core, forming a relationship of solidarity, cooperation, trust, participation, and interaction between them [61]. According to the sense of community theory, more frequent and positive interaction can strengthen the emotional connection between community members [62], community members are supported by mutual connection and common feelings [62–64], and community members feel a sense of belonging and importance to each other when common commitments are met [64]. Personalized communication and exchange play a key role in strengthening the sense of community and loyalty [65]. However, studies suggest that characteristics such as access to fresh organic food rather than a sense of community influenced some participants to participate in civic farming [21,66].

### 2.3 Consumer behavior theory

Behavioral theories are widely used by scholars and practitioners to understand consumer decision-making processes [67,68]. Previous studies have shown that attitude is an important factor that triggers customer behavior intention and thus produces actual behavior [69]. However, some studies have shown that consumers’ positive attitudes toward organic food are not necessarily reflected in their purchase behavior, and there is an attitude-behavior gap [70–75]. Behavioral intention refers to the consumer’s readiness to perform a particular behavior, including both positive and negative attitude outcomes [76], and it predicts whether the consumer will continue to purchase products from the company [77]. The concept of behavioral intention is often used in marketing due to its ability to predict customer intention. Researchers describe loyalty characteristics as behavioral intentions, which include multiple attributes such as customer repeat purchase intention, word-of-mouth, and future intention [78]. Good behavioral intentions can prompt customers to buy again or spread positive word of mouth, which will contribute to CSA’s profit. Although many studies argue that intention and attitude loyalty measures do not necessarily accurately predict subsequent behavior [79,80], the extended theory has a higher utility in explaining human behavior [81]. Therefore, this study will continue to explore consumer behavior in CSA based on behavioral theory.

### 2.4 Consumers’ experiences and retention intentions

Customer experience is seen as central to the customer’s interaction with other participants in the wider ecosystem while emphasizing the customer’s role in co-building experiences [9,82–84]. CSA maintains the direct interaction between consumers and producers, and this relationship mechanism enhances the interaction between consumers and farmers, making it more mutually beneficial and close [51]. From a consumer-farmer relationship perspective, being able to make consumers feel that they are truly involved in the activity makes their consumption experience more meaningful and encourages them to translate their participation into more sustainable practical actions [85]. A positive customer experience can increase customer loyalty [86–88]. Increasing the loyalty of members has become a key aspect of CSA’s future development [89]. Retaining existing members reduces re-recruitment efforts compared to attracting new consumers to CSA [89]. Thus, one of the major challenges for CSA farmers is how to sustain active membership participation [12]. Not only does high retention make CSA farmers more entrenched in the market, but it also reflects a satisfied group of members who are likely to recommend the farm to others, creating potential conditions for farm profitability [12]. The literature on CSA member retention intentions has largely focused on the perspectives of former members, using surveys and interviews to understand their reasons for leaving [15,17,40,53,90–94].

### 2.5 Hypotheses development

#### 2.5.1 Product satisfaction

Understanding customer experience requires measuring customer attitudes and perceptions, and customer satisfaction is an important evaluation of customer experience [95]. A key criterion for assessing customer satisfaction is the quality of the product and the quality of the services provided, which are indispensable elements for improving the performance of the company [96]. Agricultural products of similar quality are often perceived by consumers as similar products [97]. Therefore, enterprises should try to convince customers that the products they offer are among the best quality among a wide range of similar products [98]. Previous research has shown that dissatisfaction with share, especially regarding product diversity and customization options, is the main reason why former members leave [40]. The number of product varieties available has a significant impact on the continued participation of members in CSA [37,45,46]. Based on previous studies showing that product mix and lack of diversity affect membership, Galt et al. conducted a study on share customization. The results of Galt’s study showed that share customization did not lead to a high retention rate, so he proposed the customization paradox [40]. In addition to the influence of product categories and product composition on member experience, in the study of Feagan and Henderson, some members also questioned the quality and value of products, which also led them not to renew their membership [53]. Lang examined factors affecting CSA member satisfaction and showed a positive relationship between member satisfaction and support for alternatives to traditional agricultural practices (such as CSA), frequency of member farm visits, years on community farms, and the likelihood that individual shares meet their agricultural needs [20]. Evidence from China shows that CSA focusing on controlling product quality can improve consumer satisfaction with products due to food safety issues [37,99].

H1. *Product Satisfaction* has a positive effect on retention intentions.

#### 2.5.2 Interaction

Effective interaction is critical for consumers to know and understand the goals of CSA. It helps members build long-term relationships and develop loyal consumers [19,85]. DeLind emphasized that in addition to meeting the food needs of members, it is also necessary to truly integrate them into the relationship of CSA and deepen their understanding of the concept of community [5]. All participants are actively involved in various activities to enable consumers to truly understand the food production model and see themselves as part of the CSA as a whole [85]. This kind of deep participation prompts consumers to change their views in the process of interaction, to be more inclined to sustainable consumption patterns, and enhances their willingness to continue to participate [100,101]. For some CSA members, interacting with other subscribers is key to their CSA experience [19]. Research in Norway shows that members who are more actively involved in farm activities have a higher evaluation of the farm, and their overall motivation for re-participating in the farm is higher [102]. In China, members’ online participation has a positive impact on social interaction, which also positively affects the satisfaction of CSA services [29]. Some researchers believe that if interaction is not emphasized and CSA members are only regarded as customers who buy products, the foundation of CSA may be damaged [5]. However, other research results show that the main reason for members to join CSA is to obtain fresh, local, and seasonal agricultural products, and they are not interested in interaction. In the actual production process, the interaction between farm operators and members is often limited [21].

H2. *Interaction* has a positive effect on retention intentions.

#### 2.5.3 Trust

According to the shared understanding, trust is a psychological state that involves a willingness to be vulnerable based on positive expectations about the intentions or actions of others and is strengthened when the expected behavior is indeed demonstrated [103]. Providing consumers with easily accessible, understandable, and food-related information is essential to foster trust in the food system and promote sustainable consumption choices [104]. Peterson’s research shows that trust attributes have a more significant impact on the preferences of Community Supported Agriculture (CSA) consumers than other characteristics affecting consumers [27]. Based on the concern of Chinese consumers about food safety, New Rural Reconstruction (NRR) has started to promote a Participatory Guarantee System (PGS), which encourages consumers and producers to directly participate in verifying product quality to enhance consumer trust [51].

H3. *Trust* has a positive effect on retention intentions.

#### 2.5.4 Environment motivation

Previous studies have investigated the impact of local food systems on economic growth, environmental sustainability, and public health [105–109]. Organic farming and community-supported agriculture (CSA) have emerged as potential solutions to the environmental challenges associated with agricultural production [51,110,111]. Customer experience is a multidimensional construct that includes cognitive, emotional, behavioral, sensory, and social components [112,113]. Studies have shown that environmental and social trust attributes are important factors driving families to participate in CSA, and families with more sensitive perceptions of environmental issues are more likely to participate in CSA [25,114]. The study of Colorado in 2012 also showed that the motivation of consumers to participate in CSA was mainly environmental, followed by health motivation [28].

H4. *Environmental Motivation* has a positive effect on retention intentions.

#### 2.5.5 Convenience

For most consumers, CSA is a complementary channel to purchase agricultural products, and stores and supermarkets are still the first choice for consumers to purchase food because stores are more diverse and convenient [23]. Consumers who are willing to purchase CSA products are plagued by long travel times and distances and limited product choices, which cause them inconvenience [16,27]. In addition to the travel cost of purchase, the time cost of food cooking is also considered to be one of the convenience aspects, and members often need to adjust their eating habits to match the unfamiliar produce received [13,40,115,116]. But for others, spending time learning to cook unfamiliar ingredients is a form of leisure and they are willing to accept the inconvenience [117]. In this study, convenience is taken as an aspect to explore.

H5. *Convenience* has a positive effect on retention intentions.

## 3. Methods

**Fig 1:**
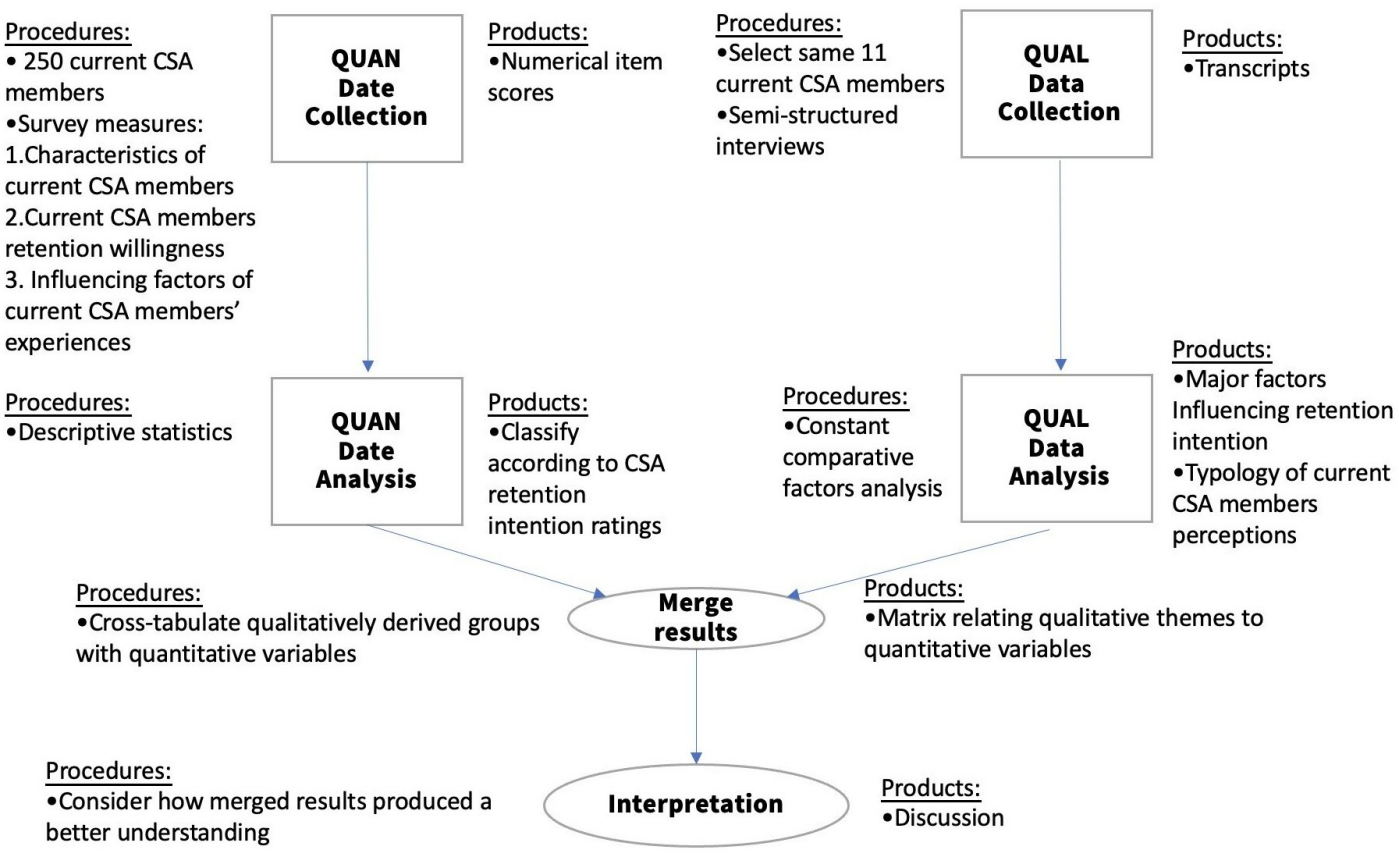
Study flow chart.

This study uses a convergent parallel hybrid research approach. The convergent parallel hybrid approach is to specify that quantitative and qualitative data are collected simultaneously, analyzed independently, and finally, the results of the study are integrated for interpretation [118]. In convergent parallel hybrid methods, qualitative and quantitative methods have no priority, and both have the same weight during data integration [119]. In parallel data analysis, the two data sets are analyzed independently until the complete analysis of the two data sets is completed. In the interpretation stage, the integration of the two data sets will produce a more complete insight into the research question [120]. We present the integration process of quantitative and qualitative research in Fig 1. With the metaphor of triangulation, we can see that integrating quantitative and qualitative data helps to get complementary results, convergent results, divergent results, or produce theoretical propositions [121]. The priority of data in the analysis of parallel studies depends on factors such as the purpose of the study [118]. In this study, both were weighted the same when data were collected, and qualitative data were placed in the context of quantitative data during analysis so that quantitative investigation was complemented by qualitative methods and further explored. Quantitative data alone is not enough to explain user experience well. Qualitative results can deduce complementary information and explain the process, to better understand the complex factors and potential reasons leading to experience. The convergence results can help to mutually verify the results of both, and the divergence results can help to improve the direction of theoretical research in the future.

### 3.1 Study 1: Quantitative strand

#### 3.1.1 Measuring tool

This study used quantitative methods and web questionnaires to collect data on the retention intentions of existing members. The questionnaire used in this study was developed based on already published research, which we reviewed in Section 2.3 to measure current customer participation intention through five reflective dimensions. Five items of product satisfaction adapted from the study of Hvitsand and Galt, et al. [40,102]. Following the research of Zoll et al., and Pole and Gary five items of interaction were chosen [21,104]. The study picked 5 items for trust from the studies of Zoll et al. [104]. The four items of environmental motivation were adapted from the study of Shelton, Hvitsand [28,102], while the five items of convenience were extracted from the study of Chen, et al., Shi, et al., Baudouin [23,122,123]. According to the study of Baudouin [23], customers’ willingness to retention intention was consulted. All measurement items of the above structure were rearranged according to previous studies. Responses were scored on a 5-point Likert scale (from 1 = strongly disagree to 5 = strongly agree), which was used to linearly assess respondent attitudes [124]. These dimensions are used to measure different aspects that influence the structure of the customer’s intention to update. The results of MacKenzie et al. provide researchers with a means of testing and evaluating dimensions and that multiple measures are the best way to ensure that a measure effectively and reliably represents a structure of interest [125]. We integrated these five dimensions following the guidelines for structural formative indicators provided by MacKenzie et al. The measurement scale is provided in S1 Table in S3 File.

SPSS 26.0 software was used to analyze the quantitative data and evaluate the econometric and structural models. In this study, we employed Multiple Linear Regression (MLR) for data analysis. MLR, a statistical method commonly used in predictive analysis, constructs a linear model involving multiple explanatory variables and a response variable. It elucidates the linear relationship of the dependent variable using ordinary least squares (OLS) regression [126]. MLR’s extensive application in customer loyalty-related analyses is well-documented. For instance, Pan et al. combined MLR with meta-analysis to explore customer loyalty predictors [127], while Kim et al. applied MLR to examine factors related to retail formats, customer satisfaction, loyalty, and demographics. Given its widespread use and suitability for diverse analyses, MLR was deemed apt for our research. In this study, the suitability for exploratory factor analysis was tested by Kaiser-Mayer-Olkin’s measure and Bartlett’s test [128]. We tested the data’s normal distribution using P-P, Q-Q plots, skewness, and kurtosis values, followed by a Pearson correlation coefficient test and a collinearity diagnosis. To mitigate the impact of data with collinearity tendencies, we utilized a stepwise forward regression method, enhancing data accuracy. The results of these tests, including P-P and Q-Q plots, are detailed in S2 Fig in S3 File.

#### 3.1.2 Sample

The study was conducted between January and March 2023. Data were collected from five CSA farms in China. It has been approved by the Kookmin University Bioethics Committee. Written consent has been obtained. Our study uses and extends the sampling framework for CSA farms provided by Chen [129]. These farms are mainly located in first—and second-tier cities in China. We ended up with five farms (Beijing Shared Harvest Farm, Guangdong Green Finger Farm, Shanghai Jiushan Agricultural Planting Farm, Fujian Jiamei Farm, and Zhejiang Zha Grandpa Organic Farm). These CSA farms are located in the north, southeast, and south of China, respectively, and their operating conditions are relatively good to some extent, which provides suitable conditions for exploring the ordering intentions of existing members. In this study, we adopted a non-probabilistic sampling method [130]. It is possible to make statistical inferences about entire groups of interest from non-probabilistic data [131]. Start with the initial "seed" sample and invite them to participate in the survey and spread the survey through their contacts. Although the results of non-probabilistic sampling cannot be considered accurate estimates, they are still indicative because almost all non-probabilistic samples contain some degree of natural randomization [132]. Therefore, our research is considered exploratory and does not claim to be representative of all CSA members in China.

We contacted the above five CSA farms with an email contact invitation to forward the online survey to members. The online questionnaire, distributed via WeChat, is open to respondents from January to March 2023 and uses limited questions to screen potential respondents. A total of 257 questionnaires were collected in this study, and 250 cases were used for follow-up analysis after completeness screening. Respondents chose "members with less than three months of experience", which was shelved. Informed consent was obtained from all participants using an ethical approval questionnaire. Pilot tests were conducted to ensure the accuracy and efficiency of data collection, and the distribution and completion of questionnaires were closely monitored to ensure their effectiveness and organization.

### 3.2 Study 2: Qualitative strand

#### 3.2.1 Participants

We used a random sampling strategy to select existing CSA members to participate in the interviews. The study started with an online search on social media platforms, Looking for members from five target farms (Beijing Shared Harvest Farm, Guangdong Green Finger Farm, Shanghai Jiushan Agricultural Planting Farm, Fujian Jiamei Farm, and Zhejiang Zha Grandpa Organic Farm), who spontaneously shared posts about their CSA farm experience on social platforms. We sent invitations to 15 members by email and/or chat functions provided by the social platform, and 11 of them agreed to participate in the interview. The 11 members were between 25 and 40 years of age, including five males and six females, and met the inclusion criteria to be existing members who had been members of CSA for more than three months. Participants were contacted via WeChat phone to schedule an appointment, and the interview session began as planned. Informed consent procedures were followed, and before each interview, the study objectives and timing were explained, participants were asked if they were current members and willing to participate, and the authenticity of farm membership was confirmed to participants. A consent form was signed by all respondents.

#### 3.2.2 The interview processes

A series of semi-structured personal interviews were conducted between February and April 2023, the interviews for this study were conducted by a single researcher to ensure consistency in interview techniques, enhancing the reliability of the data collected [118]. To further bolster the study’s credibility and address potential biases, another researcher supervised the interviews and was responsible for recording them. This approach aligns with the interpretative model of unbiased research in qualitative methods, recognizing the importance of understanding how different contexts and interviewer-interviewee dynamics can shape participants’ responses, thus providing a richer, more nuanced understanding of the subject matter. In addition, to ensure their quality and relevance, the interview questions went through a comprehensive review process. This process incorporated valuable insights and perspectives from two academic experts and agricultural researchers. The average length of each interview was 30 minutes and was conducted using an instant messaging WeChat video and virtual meeting platform (Zoom Cloud Meetings). The questions were designed according to the research focus and supported by related research [27,102].

The interview questions were based on previous research (e.g., Shelton D., 2012; Christine Hvitsand, 2016; Felix Zoll, et al., 2022) [28,102,104]. Participants were first asked to describe in detail the CSA experience they shared on their social platform (e.g., how would you rate the CSA farm event you attended?). Next, they were asked how they learned about the farm, why they joined this farm, how they evaluate CSA products, how they buy produce other than CSA, and what problems they encountered throughout the experience. Respondents were encouraged to recall moments of emotional upheaval [133], such as excitement, conflict, or surprise, and how this triggered their behavior and influenced their choices. To collect spontaneous opinions and avoid potential bias, we asked 11 participants open-ended questions about their plans to continue to join CSA in the future. After the interview, each participant received a gift certificate for a drink discount in appreciation of their contribution. To maintain ethical standards of data collection, each interview was recorded for subsequent content analysis unless requested by the respondent, and these recordings were deleted immediately after the project. Each interview lasted approximately 40 to 60 minutes. The data reached saturation after the final interviews yielded no new information.

#### 3.2.3 Analysis method

Inductive logic methods in thematic analysis were used: familiarize yourself with data, generate initial code, search for topics, review topics, define topics and name topics, and generate reports [134]. These included the following steps: First, to ensure accurate recording, all recordings were familiar and corrected by the three researchers. Then listen to the recording, read the transcript and the field notes of the assistant interviewer during the interview, and use the handwritten notes to fill in the information gaps in the recording. Interviews were conducted in Chinese, transcribed verbatim and then retroactively translated into English to ensure consistency of meaning. Three researchers independently generated the initial code by repeatedly reading the data. These codes were subsequently refined and classified, and these codes were aggregated and labeled appropriately. The highly consistent coded categories generated separately are then discussed and classified into topics [135]. Three researchers discussed the results of their thematic analysis, conducted comparisons and contrasts to identify themes and thematic frameworks, and several iterations were conducted. We then used NVivo Pro 12.0 software to upload the transcripts and systematically assign nodes and children to each text to further refine the thematic framework. Next, before generating an academic report on the analysis, the review selected examples of clear and persuasive excerpts, assessed and discussed the analysis concerning the research questions and literature in conjunction with the research objectives, and created tables by core themes, using themes, subthemes, and illustrative citations to show the range of participant responses. The tables were finally reviewed and revised to ensure an accurate and clear presentation of the data.

Researchers support the application of various methodological strategies to demonstrate the importance of qualitative rigor [136]. Strategies commonly used by qualitative researchers include audit trails, membership checks, peer debriefing, and rich descriptions [137,138]. This study assessed the credibility of the hypotheses through interdisciplinary deliberative discussions and workshop presentations conducted by the research group and reviewed the topics in collaboration with other authors to ensure representative results. In addition, triangulation was used to enhance the validity of the findings [139], using the support of quantitative research to discuss the results of data analysis and peer review, and finally to add a more explanatory section at the end of the descriptive results in conjunction with the reflection process.

## 4. Results

### 4.1 Study 1: Quantitative findings

#### 4.1.1 Participants

To conduct the questionnaire, two graduate students were recruited as surveyors, and questionnaires were administered to participants on five farms. Before participants answered the questionnaire, we asked them if they were current CSA members. Non-current CSA members were excluded. A total of 262 questionnaires were distributed, 257 were returned, and 7 invalid questionnaires were excluded. Finally, 250 valid questionnaires were collected. The total sample consisted of N = 250 members, of which 52.4% were female. In terms of age distribution, most of them were concentrated in 30–44 years old (n = 100, 40%), followed by 18–29 years old (n = 93, 37.2%). Many respondents reported having a bachelor’s degree (n = 103, 41.2%). In terms of monthly income, the number of people with monthly income between 5,000 and 8,000 was the largest (63.6%). S3 Table in S3 File shows the demographic information of these 250 participants.

#### 4.1.2 Measurement assessment

*Kaiser-Meyer-Olkin and Bartlett’s Test*. We conducted a validity test for the validity of the project. The results showed that the Kaiser-Meyer-Olkin (KMO) value was 0.947, exceeding 0.5, and the significance was less than 0.05, which proved that this data was suitable for factor exploration analysis to investigate the validity (Table 1).

**Table 1:** KMO and Bartlett’s Test.

Kaiser-Meyer-Olkin Measure of Sampling Adequacy.		0.947
Bartlett’s Test of Sphericity	Approx. Chi-Square	7737.653
	df	378
	Sig.	0.000

*Communalities*. Communalities are used to confirm whether the extracted common factors can reflect the information content of the original index. When the extracted value is greater than 0.4, it can be considered a good representation of the information content of the original index, and the subsequent analysis can be continued [140]. The results show that the Extraction value of each item is more than 0.7, indicating that the extracted public factors have reflected more than 70% of the information of the original variables, and the effect of factor analysis is good. The results are presented in the S4 Table in S3 File in the Supporting Information.

*Total variance explained*. After conducting KMO (Kaiser-Meyer-Olkin) and Bartlett’s Test and the common factor variance test, we know that the factor analysis is appropriate for this analysis. In this case, it is necessary to extract principal components to determine how many classes the original indicators can be divided into. The initial eigenvalue needs to be greater than 1 and the cumulative contribution needs to be as close to 1 as possible (close to or greater than 80%). It makes sense to require both the eigenvalues and the cumulative values. It can be found from the table that, firstly, from the point of view of the feature values, there are 6 values greater than 1, and the corresponding variance percentages reach 16.116, 15.676, 14.930, 14.470, 11.510, and 10.846 respectively. The cumulative contribution rate value is 83.548, which shows that both the feature value and the cumulative contribution rate meet the requirements. Therefore, all the original indicators can be classified into six categories. The results are presented in the S5 Table in S3 File in the Supporting Information.

*Rotated Component Matrix*. The Rotated Component Matrix was used to classify the data. It can be found from the table below that the load of the tested items on a single dimension is higher than 0.5, so they are valid items and pass the validity test to retain items. On factor 1, larger loadings of Q26, Q27, Q28, Q29, and Q30 can be divided into one latitude. Similarly, the latitude division is shown in Table 2. These indicators are consistent with the original dimension division. The scale items are presented in S1 Table in S3 File.

**Table 2:** Rotated Component Matrix^a^.

	Component					
	1	2	3	4	5	6
PS1Q7	0.182	0.119	0.203	**0.788**	0.140	0.122
PS2Q8	0.227	0.198	0.212	**0.798**	0.162	0.090
PS3Q9	0.242	0.119	0.193	**0.795**	0.130	0.185
PS4Q10	0.198	0.147	0.145	**0.776**	0.174	0.224
PS5Q11	0.252	0.164	0.266	**0.720**	0.131	0.226
I1Q12	0.100	0.230	**0.707**	0.344	0.320	0.185
I2Q13	0.147	0.253	**0.808**	0.267	0.164	0.185
I3Q14	0.163	0.250	**0.816**	0.219	0.204	0.164
I4Q15	0.159	0.263	**0.830**	0.193	0.207	0.224
I5Q16	0.140	0.298	**0.796**	0.184	0.197	0.194
T1Q17	0.133	**0.846**	0.227	0.153	0.177	0.185
T2Q18	0.211	**0.799**	0.242	0.176	0.119	0.179
T3Q19	0.177	**0.786**	0.248	0.151	0.241	0.171
T4Q20	0.201	**0.818**	0.218	0.147	0.190	0.187
T5Q21	0.201	**0.815**	0.231	0.144	0.197	0.169
EM1Q22	0.231	0.235	0.260	0.217	**0.768**	0.109
EM2Q23	0.226	0.238	0.254	0.194	**0.812**	0.146
EM3Q24	0.209	0.249	0.201	0.144	**0.778**	0.250
EM4Q25	0.284	0.184	0.234	0.203	**0.705**	0.243
C1Q26	**0.846**	0.156	0.140	0.258	0.185	0.213
C2Q27	**0.795**	0.194	0.173	0.239	0.215	0.152
C3Q28	**0.864**	0.164	0.117	0.212	0.173	0.191
C4Q29	**0.864**	0.162	0.104	0.218	0.184	0.165
C5Q30	**0.810**	0.248	0.137	0.199	0.172	0.210
R1Q31	0.234	0.238	0.254	0.219	0.197	**0.773**
R2Q32	0.293	0.174	0.163	0.264	0.196	**0.725**
R3Q33	0.253	0.291	0.295	0.222	0.223	**0.690**
R4Q34	0.239	0.273	0.232	0.213	0.173	**0.779**

^a^ *PS*: Product Satisfaction, *I*: Interaction, *T*: Trust, *EM*: Environmental Motivation, *C*: Convenience, *R*: Retention Intention.

*Reliability statistics*. For the questionnaire, the reliability test is mainly determined by the value of Cronbach α coefficient. When the coefficient value is greater than 0.8, the reliability is very good. When the coefficient value is lower than 0.6, the items need to be added or deleted. The results show that the total Cronbach α coefficient value is above 0.9, 0.968, and the reliability value of each sub-dimension is also above 0.9, so the reliability is up to the standard. The correction for the total correlation is also greater than 0.7, so we don’t need to remove it Table 3).

**Table 3:** Reliability test.

Reliability Statistics (Cronbach Alpha)				
Items	Corrected Item-Total Correlation(CITC)	Cronbach Alpha if Item Deleted	Fractal dimension	Cronbach α
PS1Q7	0.767	0.912	0.923	0.968
PS2Q8	0.822	0.901		
PS3Q9	0.824	0.9		
PS4Q10	0.798	0.905		
PS5Q11	0.786	0.908		
I1Q12	0.836	0.956	0.958	
I2Q13	0.891	0.946		
I3Q14	0.886	0.947		
I4Q15	0.923	0.941		
I5Q16	0.874	0.949		
T1Q17	0.889	0.937	0.952	
T2Q18	0.848	0.944		
T3Q19	0.853	0.943		
T4Q20	0.873	0.94		
T5Q21	0.871	0.94		
EM1Q22	0.829	0.908	0.928	
EM2Q23	0.887	0.888		
EM3Q24	0.829	0.908		
EM4Q25	0.786	0.922		
C1Q26	0.922	0.95	0.963	
C2Q27	0.857	0.96		
C3Q28	0.915	0.951		
C4Q29	0.907	0.952		
C5Q30	0.875	0.957		
R1Q31	0.865	0.896	0.929	
R2Q32	0.784	0.923		
R3Q33	0.822	0.911		
R4Q34	0.863	0.897		

^a^ *PS*: Product Satisfaction, *I*: Interaction, *T*: Trust, *EM*: Environmental Motivation, *C*: Convenience, *R*: Retention Intention.

#### 4.1.3 Multiple linear regression analysis

*Test for normal distribution*. According to the requirements of Pearson and multiple linear regression in statistics, all variables must meet the criteria of normal distribution. Therefore, P-P, Q-Q, and kurtosis skewness tests were used for data review. The results are normally distributed, and P-P and Q-Q are provided in S2 Table in S3 File in the Supplementary Appendix. According to the study, the distribution of data is in line with the normal distribution when the skewness value is less than 3 and the kurtosis value is less than 9 [141]. Firstly, from the perspective of skewness and kurtosis value of the items, the absolute value is less than 3, so it can be considered that the items are in line with normal distribution. Secondly, from the perspective of the mean value, they are all around 3, which is relatively at a medium level and has room for improvement (Table 4).

**Table 4:** Normal distribution test descriptive analysis of different items.

Items	n	Mean	Std.	Skewness	kurtosis
PS1Q7	250	3.352	1.096	-0.347	-0.473
PS2Q8	250	3.408	1.015	-0.212	-0.551
PS3Q9	250	3.472	1.05	-0.22	-0.645
PS4Q10	250	3.388	1.013	0.051	-0.642
PS5Q11	250	3.344	1.08	-0.064	-0.588
I1Q12	250	3.12	1.03	0.068	-0.248
I2Q13	250	3.06	0.99	0.229	-0.115
I3Q14	250	3.128	1.006	0.289	-0.279
I4Q15	250	3.1	1.007	0.178	-0.229
I5Q16	250	3.004	1.036	0.298	-0.197
T1Q17	250	2.972	1.081	0.094	-0.488
T2Q18	250	3.016	1.068	0.167	-0.479
T3Q19	250	2.952	1.029	0.164	-0.24
T4Q20	250	2.976	1.025	0.341	-0.381
T5Q21	250	2.924	1.082	0.267	-0.383
EM1Q22	250	3.308	1.009	-0.104	-0.337
EM2Q23	250	3.152	1.042	0.164	-0.624
EM3Q24	250	3.176	1.01	0.182	-0.378
EM4Q25	250	3.352	1.036	-0.113	-0.349
C1Q26	250	3.508	1.061	-0.173	-0.591
C2Q27	250	3.496	1.091	-0.205	-0.809
C3Q28	250	3.408	1.12	-0.113	-0.675
C4Q29	250	3.468	1.08	-0.09	-0.771
C5Q30	250	3.408	1.08	-0.059	-0.662
R1Q31	250	3.144	1.066	0.131	-0.427
R2Q32	250	3.212	1.071	0.121	-0.575
R3Q33	250	3.24	1.075	0.037	-0.487
R4Q34	250	3.172	1.063	0.116	-0.53

^a^ *PS*: Product Satisfaction, *I*: Interaction, *T*: Trust, *EM*: Environmental Motivation, *C*: Convenience, *R*: Retention Intention

**Fig 2:**
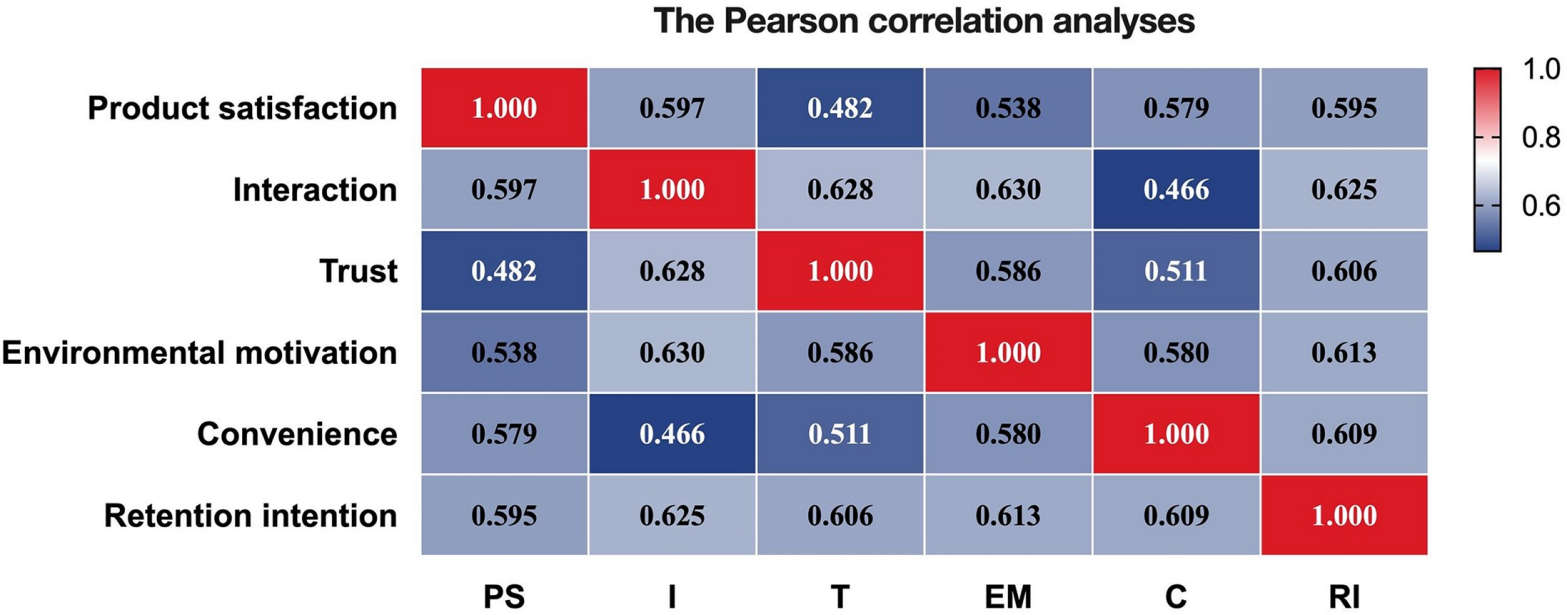
Pearson correlation analyses. a *PS*: Product Satisfaction, *I*: Interaction, *T*: Trust, *EM*: Environmental Motivation, *C*: Convenience, *R*: Retention Intention.

*Pearson correlation*. Pearson correlation was used to assess the degree of correlation between the five influencing factors and user retention intention [142]. Through the correlation analysis, it can be found that the absolute value of the correlation coefficient between product satisfaction, interaction, trust, environmental motivation, and convenience is less than 0.8, so it can be considered that there is no collinearity problem between the independent variables. Secondly, from the correlation coefficients between product satisfaction, interaction, trust, environmental motivation, convenience, and retention intention, all five factors are positively correlated with retention intention, and the hypothesis is initially established (Fig 2).

*Multiple linear regression of stepwise method*. The stepwise regression method uses a reasonable screening mechanism for independent variables, which helps to exclude independent variables that do not have a statistically significant effect on the regression equation [143]. The method progressively adds parameters to test their effect on the model, subsequently retaining the filtered variables related to the dependent variable and excluding other irrelevant variables (S6 Table in S3 File).

The results show that the R^2^ of the final comprehensive model of "interaction", "convenience", "trust", "product satisfaction" and "environmental motivation" is 0.576 and P < 0.01. It shows that the five independent variables can explain 57.6% of the variation of the dependent variable, that is, 57.6% of the dependent variable’s retention intention is affected by the five independent variables. The significance of the regression model was examined by ANOVA. The result of this data shows that F = 66.204, P<0.05, indicating that the regression model is significant.

In the final model results, the variables "interaction", "convenience", "trust", "product satisfaction" and "environmental motivation" are retained in the stepwise model. These five variables and "willingness to retain" constitute the functions in this model relationship. The partial regression coefficient for each variable included in the model is nonzero, and the P for each independent variable is less than 0.01. The final regression model of the stepwise method is as follows: Retention Intention = 0.102 + 0.211 Interaction + 0.224 Convenience + 0.193 Trust + 0.176 Product Satisfaction + 0.152 Environmental Motivation.

The standardized regression coefficient Beta is used to compare the effects of different independent variables on the dependent variable. Under the premise of statistical significance, a larger standardized regression coefficient Beta indicates a larger influence effect of the corresponding independent variable. In this case, under the premise of satisfying statistical significance, the order of the impact on retention intention is convenience, trust, interaction, product satisfaction, and environmental motivation. Product satisfaction can significantly positively affect retention intention. The regression coefficient is 0.176 > 0, and p < 0.01, which means that every 1 increase in product satisfaction will lead to a 0.176 increase in retention intention. The results of other variables are shown in Table 5. A low VIF value (< 3) indicates that the final five independent variable observations chosen to model are free of multicollinearity issues and have significant explanatory power in explaining the maximum model variation.

**Table 5:** The results of linear regression analysis (N = 250).

Items	Unstandardized Coefficients		Standardized Coefficients	t	p	VIF
	B	Std. Error	Beta			
Constant	0.102	0.177	-	0.577	0.564	-
Product satisfaction	0.176	0.061	0.167	2.895	0.004**	1.912
Interaction	0.211	0.065	0.204	3.26	0.001**	2.25
Trust	0.193	0.058	0.193	3.329	0.001**	1.924
Environmental motivation	0.152	0.063	0.146	2.402	0.017*	2.12
Convenience	0.224	0.054	0.234	4.135	0.000***	1.845
R^2^	0.576					
Adj R^2^	0.567					
F	F (5,244) = 66.204, p = 0.000					
D-W	1.819					

Dependent Variable: Retention intention.

Note: ^1^* p<0.05 ** p<0.01 *** p<0.001.

Thus, hypotheses H1, H2, H3, H4, and H5 are supported.

*Out-of-sample testing*. This study conducted in-sample training and out-of-sample testing with a sample size of 7:3. Due to the sample size of 250 for in-sample training, an additional 108 pieces of data were collected for out-of-sample testing in this study. The out-of-sample testing in this study consists of two parts: (1) testing of out-of-sample predictive ability, and (2) robustness analysis of regression coefficients.

In terms of out-of-sample predictive ability testing, the prediction equation derived from the in-sample training is: retention intention = 0.102 + 0.176 * product satisfaction + 0.211 * interaction + 0.193 * trust + 0.152 * environmental motivation + 0.224 * convenience level. According to this equation, the retention intention of the 108 data samples was predicted, resulting in an average absolute error (Mean Absolute Error, MAE) of 0.3997 and an average absolute percentage error (Mean Absolute Percentage Error, MAPE) of 16.89%, indicating good prediction accuracy.

When it comes to the robustness analysis of regression coefficients, multiple linear regression analysis was conducted with product satisfaction, interaction, trust, environmental motivation, and convenience as independent variables, and retention intention as the dependent variable. The results are shown in Table 6. The goodness of fit of the model reached 0.618, and the DW test and VIF collinearity test passed. The regression coefficients of the five independent variables are all positive and significant at a significance level of 0.05. In terms of the degree of impact, trust, interaction, convenience, product satisfaction, and environmental motivation are ranked in descending order. The high consistency between the out-of-sample regression results and the in-sample regression results based on 250 samples proves the robustness of the regression coefficients.

**Table 6:** Out-of-sample regressive analysis (n = 108).

	Unstandardized Coefficients		Standardized Coefficients	t	p	VIF
	B	Std. Error	Beta			
Constant	-0.316	0.295	-	-1.071	0.287	-
Product satisfaction	0.206	0.082	0.203	2.523	0.013*	1.724
Interaction	0.251	0.085	0.241	2.941	0.004**	1.792
Trust	0.255	0.076	0.266	3.363	0.001**	1.668
Environmental motivation	0.172	0.079	0.147	2.185	0.031*	1.209
Convenience	0.203	0.077	0.208	2.622	0.010*	1.679
R 2	0.618					
Adj R 2	0.599					
F	F (5,102) = 33.017, p = 0.000					
D-W	1.212					

Dependent Variable: Retention intention.

* p<0.05

** p<0.01

*** p<0.001.

### 4.2 Study 2: Qualitative findings

Four themes in CSA participation influence a member’s willingness to continue to participate. These themes were Product quality, Customization, Offline experience, and Online services.

These themes focused on expressing how members felt after participating and how the various services offered by the farm influenced the user’s mindset and behavior toward participation. These 4 themes cover the positive experiences members had during their experience, their motivation to continue participating, the problems they encountered during their participation, and the services they would like to see improved during their experience. These 4 themes are presented in Table 7.

**Table 7:** Emergent themes, definitions, and example quotations.

Theme	Sub-theme	Example
Product quality	The freshness of the food’s appearance The taste of the food The integrity of the product’s packaging.	Meat is fresh, and when I cook it, well, it’s dense, and tender. And I think it’s better than which is sitting out for a long time or has been frozen for a long time. (P.4). My family recognizes its quality. It tastes very good. In terms of flavor, it does have a more vegetable flavor.… That’s when you first feel that the plant is a little bit more like a plant. There is no excessive additive taste. (P.10). …and the taste of this food is better and fresher than what I bought in the supermarket before … I think this is healthier. (P.9). The vegetable box I received contains rice, vegetables, and some meat. It is all opened separately. I think the preservation is also done well. (P.9).
Customization	Food Variety choices The ratio of the types can be selected.	I prefer food like potatoes or carrots, I can tell him that and then he will adjust it with me in terms of proportions. So, it’s a very user-friendly service now. (P.3). For the products they will deliver recently, they will have a form in advance and send it to me in the form of pictures. On this form, you can check what you want and what you don’t want. (P.9).
Offline experience	Members brought their families to the event. Experienced the good service from the staff. The beautiful surroundings Good leisure programmed. Positive food education for children.	The last event I attended was with my family. My parents are more interested than me. (P.8). The staff will teach us how to feed the rabbits and how much to feed. I think their guidance is detailed, and they also remind us to pay attention, and to pay attention to safety when doing it by ourselves, so as not to get hurt. (P.4). The area of this farm is very large, it is a bit like a garden landscape. The scenery is very beautiful. I think the environment is very suitable for retirement. (P.9). If given the chance, I would take part in this activity again. Because I feel that participating in these activities, people in the city now feel that life is more stressful, and I am more yearning for such a life in the countryside. (P.1). It as a free membership service. If you take your children to other farm experience, the fee is quite a lot. (P.4). We took the children to experience it, and we thought it was very fun. The child is willing to get close to the little animals and he said that it is not easy for the little rabbit to grow so big. We must cherish all the food in the future. (P.4).
Online services	Members communicate with the farm. Purchase shares. Increase trust in food safety. Farm’s promotion on social media.	I can choose the type of vegetables, and they made a small program where I can choose by myself. (P.2). I think dried fruit, compared to fresh fruit, I may not want to buy… I may be a little worried the production process is not clean. If he can be more transparent like such live streaming video to explain to consumers, I can put these steps on the bright side, I might feel more at ease. (P.8). I saw the farm’s publicity on the official account, and other social platforms as self-media publicity, and they have their own organic certificate, so this really gave me some trust. (P.10).

Product quality is the main reason why some members rejoin the CSA. He will continue to renew his membership if the farm maintains a supply of quality produce. In our interviews, we found that members judge the quality of the product in three ways: the freshness of the product that can be seen, the taste and texture, and the way the product is preserved and packaged during the delivery process. Participants indicated that the quality of the products they received made them satisfied with the farm. Members felt that the products they received were fresher than those purchased in supermarkets or other offline shops. As a result, they intend to join the farm again.

Customization consists of food variety choices and the ratio of the types can be selected. Participants’ evaluations of the customization service varied according to the farms involved, but all shared an expectation of a customizable service for products. Participants reflected that if the farms offered too small a variety of products or if they could not choose what they wanted, they would turn to other ways of buying products with more options. To the satisfaction of members, some farms offer a customizable service, where the farm provides a form where they can choose the products, they want in that delivery box. In terms of customizing product shares, the vegetable boxes offered by CSA farms are essentially organized into shares on a household basis. This would result in waste, as some members who live alone or with fewer family members would not be able to consume the vegetables they receive. In addition, there are ad hoc situations where the share cannot be adjusted promptly, such as business trips and eating out. One participant reported that they had experienced delivery errors with their tailor-made products, and while participants could accept occasional errors, they were still frustrated when they received incorrectly delivered items.

Many participants stated that participating in the experiential activities held by the farm made them more aware of the sustainable farming concept of the farm and gained many positive experiences during the activities. Members are satisfied with the activities organized by the farm and the service provided by the staff. Because of the on-farm activity experience included in membership services, many members believe that the cost of joining a CSA is worth it. The farm environment is also a point that users value. As a farm around the city, it provides a good experience environment for users to relax, relieve the pressure of urban life, and obtain a good activity experience. Members who took part with their children felt that it was not only a fun activity to take their children to the games, but also a way for the children to learn to value food more.

Online services (such as mini-programs, websites, etc.), communication platforms, and social media platforms are provided by the farm. Due to the different development scales of farms, the perfection of their online platform services is also different. While some farms already have a complete online purchase and after-sales service process in place, for others, members still need to customize varieties or contact farms by phone. According to some participants, the most important function of online platforms is to facilitate connections with farms. Some farms do not have such a communication platform, they only have a farm information release platform (WeChat public), the farm will timely release the latest information the farm, but members prefer to be able to easily contact the farm, rather than unilaterally receive the information of the farm. In terms of online social media, many members would like to see farms sharing themselves on different social platforms, such as the production process, the growing process of plants, and the production process of non-food products, to learn more about the products they receive.

### 4.3 Study 3: Mixed-methods comparison

This study used a convergent parallel design to explore the factors influencing the retention intentions of existing CSA members. Through a direct comparison of quantitative and qualitative results, the results of this study suggest that there is both convergence and divergence in the factors that influence the willingness of members to re-engage.

In the quantitative model, product satisfaction is positively related to member re-joining intention. This result converges with the qualitative results. In the quantitative model, based on the item results of product satisfaction, members generally believed that the quality of the product was in line with their expectations and considered CSA to be a good choice for purchasing organic food. In the qualitative data, participants also recognized the quality of products provided by the farm and indicated that if the product quality was stable and the membership fee did not increase much, the desire for quality products was still an important reason for them to continue to join.

In the quantitative model, trust is positively related to a member’s intention to re-participate. This result is the same as the qualitative result. Some respondents believed that witnessing the operation of the farm by participating in offline experiences and online platforms would increase their trust in the farm. Respondents believed that the farm would deliver to them products produced according to organic standards as agreed, which made them feel that products obtained through CSA were more reliable and the quality of the products was more guaranteed than those purchased through other channels. Therefore, they would prefer to remain members of CSA.

The quantitative linear results showed that convenience positively affected participants’ retention intention. This is similarly demonstrated by the qualitative findings. The respondents believe that there are too many channels for quick purchase of agricultural products, which are different from the supermarket or farmers’ market in the past. Now there are many e-commerce platforms for ordering fresh agricultural products, so speed and convenience are important factors for them to consider. At the same time, participants explained that because there are many online purchase channels for them to choose from, they may also consider not renewing their membership if CSA does not provide the same convenience.

The quantitative results show that interaction has a positive effect on the retention intention of members. This finding is consistent with the qualitative results. Interaction in the quantitative model refers to the willingness of participants to interact directly with farmers and to meet some like-minded friends on the farm. In the qualitative data, respondents stated that interacting directly with the staff of the farm helped them understand this production model and increased their sense of identity with it. At the same time, they think that this kind of interaction is more like a leisure activity for them, and participating in farming makes them relax.

In the quantitative results, concern for environmental protection is positively related to retention intention. This result is different from the qualitative result. In the qualitative results, some participants believed that they had some environmental awareness before joining the farm and used environmental measures such as biodegradable meal boxes and energy-efficient appliances in their daily lives. One participant suggested that supporting sustainable agriculture is socially beneficial. More participants indicated that they had never focused on the issue. Participants believed that their attitudes towards environmental protection did not affect whether they would choose to join CSA again.

## 5. Discussion

Importantly, we find that regarding the convenience factor, while both results are consistent, they are viewed differently in the quantitative and qualitative results. In the quantitative results, convenience is reflected in the delivery method. In qualitative research, convenience is not only reflected in the delivery but also further extended to online services. In addition to shared purchases, other categories of products can also be delivered together. Since COVID-19, online sales of agricultural products have increased significantly, and consumer demand for online food supply has been increasing [144]. Members need a more complete online purchase service system, from understanding the product and booking to after-sales service. They will inevitably be compared with other online purchasing platforms, so providing relatively perfect online services is more important for members. Participants felt that after joining the membership, they reduced the number of offline purchases of agricultural products, saving themselves time and the process of selecting products. Compared with online purchases from other channels, variety matching on CSA farms is more convenient. This is also supported by previous research showing that convenience has a positive impact on consumer participation intention [23,53]. This combination of online and offline sales not only provides convenience for members but also is closely related to the concept of multi-channel sales in retail. Previous research has shown that such multi-channel selling strategies create consumer satisfaction through the interaction between offline and online behaviors [145], enhance attitudes toward stores, improve purchase intentions [146,147], and positively impact customer retention to build strong customer relationships [148,149]. Effective offline and online collaborative management can optimize the overall customer experience [150–153]. Consumers are increasingly using smartphones to evaluate and purchase products, which requires a more integrated and cohesive customer interaction experience [154]. The business strategy in the retail field provides a reference for CSA to adapt to the digital market and enhance the member experience. In previous studies, non-customizability may cause members to receive unfamiliar ingredients and drop out of CSA due to cooking barriers [12,115,116]. The Chinese CSA farms mentioned in the interview have made customization a basic service for members, which removes the barrier of inconvenient cooking. Most of the respondents indicated that they could customize the type and proportion of produce they wanted with the farm, which they considered a very human service. Members can make comments on the order on the online platform or contact the relevant service personnel by telephone. Customized services increased the goodwill of members towards the farm [15,22,92,155].

Regarding the interaction factors, the perceptions of the participants were also complemented by a wider range of views than in the quantitative studies. In some CSA farms in China, the interaction between farmers and members is somewhat different from the original vision of CSA. The interaction emphasized in the past was to connect members more closely with farmers through their labor participation, thus forming an emotional connection. For the existing CSA farms in China, they prefer to turn this interaction into a trip to a farm near the city. This connection has evolved from a labor to a recreational nature. The interview results showed that participation in offline activities had a greater impact on the participation of families with children. Participants believed that by joining the farm, they could not only eat healthier and more organic food but also enjoy the services of a free parent-child farm experience. This is one of the motivations for them to continue to join the farm in the coming years. The results of previous studies verify the positive impact of participating in offline interactive experiences on membership renewal [26,85]. Although interactivity was the factor influencing members’ willingness to re-engage in this study, its connotation changed greatly, and whether this recreational experience can connect farmers and members more firmly than the experiential bond of participating in labor needs to be further explored.

Although environmental motivation would positively affect member retention intention in the quantitative results, this was not reflected in the qualitative results. To be sure, existing members who participated in the interviews stated that they were more environmentally conscious after joining CSA farms than before and that knowing they were doing something good for the environment and society increased their positive attitude, although this experience was not enough motivation to maintain membership. Previous studies have shown that ethical consumption such as environmental protection can provide long-term retention support [25,114]. Most members did not fully understand the concept of a CSA farm perse. They believe that there are many outlets to buy organic food and that CSA is not their only option, so they cannot continuously participate based on conceptual identification and environmentally friendly planting concepts. Members all mentioned that they had acquired relevant knowledge on the farm, and the farm had made some efforts in environmental education, but the gains were few and not enough to cause a change in the awareness of the members.

Regarding the exploration of product satisfaction and trust factors, our findings are consistent with previous studies on member experience. An affirmation of product quality will support the retention of members [21,22]. The food safety problem in China has been widely discussed by consumers [37,99]. Respondents indicated that they attached importance to food safety and health issues before joining the farm, and the quality products provided by CSA farms met their needs. Dietary perceptions have a positive impact on membership participation in CSA farms [114,156]. They mentioned that they pay more attention to food quality after participating in CSA farms. Besides CSA, they have other complementary purchase channels, and they also choose to buy organic certified food. Higher quality products and healthy organic food are the most important factors influencing members to choose to purchase and actively create more sustainable purchasing behavior [37]. After purchasing organic food, people pay more attention to dietary health information and thus buy organic food again, forming a virtuous cycle [15,94,157]. Trust increases a member’s willingness to join again [19]. The trust factor here refers to the combination of online and offline ways, which provides members with a condition that people can monitor the growing environment of food by themselves, they can control the source of food they buy, and enhance their trust in the farm. Therefore, trust in the farm is an important factor affecting member retention [27,104].

## 6. Conclusion

This mixed approach to CSA farms explores the experiences of existing members and identifies factors that influence existing members to join the farm again. This paper collects data on existing CSA members in China, and the quantitative results show that convenience, trust, interaction, product satisfaction, and environmental motivation positively influence members’ intention to participate again. The understanding of the factors influencing the intention to re-engage became more specific when insight was given to the participant’s understanding of the factors influencing the intention to re-engage. The qualitative research results found four themes product quality, customized service, online service, and offline experience. Overall, convenience is the main factor that Chinese CSA members value more and support their continued participation in CSA, followed by product quality and offline experience. The quantitative data in this study helped to identify trends and the qualitative data provided valuable insights into changes in these trends. As researchers, we aim to use theoretical and empirical evidence to develop strategies based on CSA farms and alternative farm alliances to enhance the service experience of existing members. A broader understanding of the experiences of CSA farm members will help develop more comprehensive strategies to support users continued engagement with farm activities.

### 6.1 Theoretical implications

This study employed a combined quantitative and qualitative approach to data collection in China. The quantitative findings corroborate factors identified in prior research, while the qualitative insights offer a nuanced examination of these factors, thereby broadening the scope of measurement dimensions established in earlier studies. This integration underscores the value of employing a convergent parallel design. Our results necessitate a reevaluation of the factors influencing existing members’ retention intentions. The insights gleaned from in-depth interviews present an opportunity for future research to explore these factors across various geographic and methodological contexts. Based on this study, researchers can further explore how to develop new farm operation models combined with online experience while following the CSA farmer and member cooperation concept. It is necessary to continue to verify whether adjusting CSA according to member experience is more conducive to the sustainable development of CSA in future studies.

### 6.2 Practical implications

Building on the practical implications of our research, it’s evident that product quality is pivotal for member retention. Managers should prioritize maintaining high-quality, natural, and organic products. With the surge in e-commerce, a stronger focus on online channel management is crucial to enhance member retention. Leveraging social media in tandem with online sales can deepen trust and understanding among existing CSA members while attracting new consumers.

CSA’s role in addressing climate change and promoting sustainable agriculture should be communicated through diverse channels, including online platforms and farm live broadcasts, to minimize member loss. Farmers can attract and retain members by organizing social events to spread the benefits of local production. And actively carry out farm volunteer activities to allow more consumers to participate in the farm experience. Integrating traditional farming with innovative recreational activities can enrich the on-farm experience for members. Moreover, CSA offers exciting opportunities for young farmers to apply new techniques and marketing strategies, keeping pace with contemporary trends.

Policymakers improve farmers’ knowledge of environmentally friendly agriculture by providing education programs and technical assistance. From a policy standpoint, supporting farmers’ groups financially and adopting incentives such as subsidies to support their environmentally friendly practices. Sustainable practices need to be recognized at a wider public level. Public awareness is raised through effective publicity to inform the public about the numerous benefits of CSA. Aligning agricultural practices with environmental protection, employment support, and consumer health can create a more sustainable and health-conscious food system.

### 6.3 Limitations and future research

The limitations of this study suggest that further research is needed. The experience of existing members may vary depending on factors such as specific farms, natural climates, and regional economies. While we sought to obtain a diverse sample by recruiting from a range of public Settings, it is important to note that this study focused only on farms located in eastern China. The study provides insights into specific groups of CSA farm members in eastern China and should therefore be seen as a starting point for further investigation through a larger, longer-term mixed-methods study. In addition, the framework used to measure member reengagement willingness is limited to five dimensions and is based on the scale developed by Zoll et al. [104] Although qualitative research was conducted to explore various factors, it did not reflect many other factors that influence user re-engagement intention factors. Therefore, a comprehensive study should cover more possible factors or dig deeper with a more qualitative approach, which is a suggestion for future research. Third, while this study focuses on the internal factors of the CSA model that affect member retention, the study may have overlooked external influences such as market trends, economic conditions, or policy changes. These external factors should be considered in future research, and a more comprehensive analysis could provide a more complete picture of their impact on member experience and retention intention. Fourth, asking whether a person is satisfied with the service is easily explained, and in this study, participants consider the service they experience and what they think helps or improves the experience. While the responses of the participants were very detailed and thoughtful, other factors not involved in the study were underestimated. Future research should consider expanding the scope of the study, increasing the sample size, and collecting more comprehensive user experience data. Fifth, this study provides new thinking directions for future studies of CSA consumers. Omni-channel sales or the combination of online and offline marketing methods, how to provide reference for the development of CSA new scenarios, and how to better apply to the CSA model is still worthy of further study. How CSA as a niche food system can be promoted to bring it into the public consciousness, we need to further assess consumer understanding of the value of food. This will help us find more effective strategies and channels to bring CSA into the public eye. In the future, we will continue to explore the transformative potential of CSA and promote its sustainable development in the food system.

## Supporting information

S1 File (DOCX)

S2 File (DOCX)

S3 File S1 Table. This is the S1 Table measurement. S2 Fig. This is the S2 Fig P-P, Q-Q. PS: Product Satisfaction, I: Interaction, T: Trust, EM: Environmental Motivation, C: Convenience, R: Retention, Intention. S3 Table. This is the S3 Table The demographic information of research samples in phase one (N = 250). S4 Table. This is the S4 Table Communalities. S5 Table. This is the S5 Table Total Variance Explained. S6 Table. This is the S6 Table Stepwise multiple linear regression.
(PDF)
