# Supplementary material for: Investigating consumers’ experiences with community supported agriculture: Convergent parallel design methods
Source: PLoS One. 2024 May 13;19(5):e0303184. doi: 10.1371/journal.pone.0303184 (PMC11090362; doi:10.1371/journal.pone.0303184)
Supplement: S1 File — (DOCX) [file pone.0303184.s001.docx]

| **Construct** | **Item** | **Resource of measurement** |
| --- | --- | --- |
| Product Satisfaction | PS1. The quality of my CSA produce meets my expectations. | Felix Zoll, et al., 2022  Christine Hvitsand  2015  Ryan E. Galta, et al.,2019 |
|  | PS2. The price of the harvest share is appropriate. |  |
|  | PS3. I have a better selection of organic food. |  |
|  | PS4. The produce mix offered by CSA met my needs. |  |
|  | PS5.I can customize the types and proportions of produce in the "Vegetable Box". |  |
| Interaction | I1. Interacting directly with farmers has strengthened my willingness to purchase CSA services. | Felix Zoll, et al., 2022  Antoinette Pole, et al.,2013 |
|  | I2. In my CSA there are sufficient opportunities for member participation. |  |
|  | I3. To meet like-minded people in farm is important to me. |  |
|  | I4. If I have questions, I can get answers directly from the staff on my farm. |  |
|  | I5. It is important to me to have the possibility to visit the CSA to personally get an idea of the production of my food. |  |
| Trust | T1. I generally trust in my CSA. | Felix Zoll, et al., 2022 |
|  | T2. I generally trust in the farmer(s) of my CSA. |  |
|  | T3. I believe that the products of CSA farms meet the product quality regulations set by the CSA platform. |  |
|  | T4. I trust that my CSA does not overcharge for their products.  T5. I trust my farm to uphold environmental standards during production. |  |
| Environmental Motivation | EM1. Supporting environmentally friendly practices is my motivation for joining CSA. | Christine Hvitsand  2015  Danielle del Castillo Shelton, B.S.2012 |
|  | EM2. Participation in my CSA plays an important role in improving and protecting the environment. |  |
|  | EM3. We should reduce the consumption of meat in favor of environment, health and animals. |  |
|  | EM4. To be a CSA-member is one of the most important environmental measures our household does. |  |
| Convenience Degree | CD1. I can walk to a community pickup location to pick-up my order. | Junhong Chen, et al.,2019  Ruoding Shi, et al.,2015  Baudouin Q.2010  Emily H. Morgan, et al.,2018 |
|  | CD2. I can pick-up produce on the farm with transportation. |  |
|  | CD3. I can get my products through online order and delivery service. |  |
|  | CD4. I was able to get my product just in time when I wanted it. |  |
|  | CD5. I find it easier to order at CSA than to buy produce at the supermarket. |  |
| Member Retention | MR1. I will continue to renew my CSA membership. | Baudouin Q.2010 |
|  | MR2. I would recommend to people who are important to me to join my CSA. |  |
|  | MR3. I am satisfied with the services provided by the CSA. |  |
|  | MR4. I will share my experience in CSA on social platforms. |  |

The scale ranges from 1 to 5, 1 = Strongly Agree, 2 = Agree, 3 = Neutral, 4 = Disagree, 5 = Strongly Disagree

**Demographic Survey**

1. What is your gender?

- Male

- Female

2. Please indicate your age.

- 18-29

- 30-44

- 45-59

- 60-65

- Above 65

3. How many people does your typical food bill cover?

1-one; 2-two; 3-three; 4-four; 5-five; 6-six; 7-seven; 8-eight; 9-nine or more; 10-zero

- Under 2 years of age 0-1-2-3-4-5-6

- Age 1-6 0-1-2-3-4-5-6

- Age 7-17 1-2-3-4-5-6

- Age 18-30 1-2-3-4-5-6

- Age 31-40 1-2-3-4-5-6

- Age 41-50 1-2-3-4-5-6

- Age 51 and above 1-2-3-4-5-6

4. What is your highest level of education?

- Graduate school

- Bachelor’s degree

- Some college or associate degree

- High School degree or equivalent

- Lower than High school

5. What is your monthly income?

- Less than ¥3000

- Between ¥3000 and ¥5000

- Between ¥5000 and ¥8000

- Between ¥8000 and ¥15000

- More than ¥15000

6. What is your occupation?

- Management, professional, and related occupations 行政

- Service occupations

- Sales and office occupations

- Farming, fishing, and forestry occupations

- Construction, extraction, and maintenance occupations

- Production, transportation, and material moving

- Unemployed

- Student

- Retired

- Other: ____________

7. Do you have any comments, opinions, suggestions about CSAs or more generally about food bought directly from farmers? If yes, feel free to use the space below.
