# Supplementary material for: Investigating consumers’ experiences with community supported agriculture: Convergent parallel design methods
Source: PLoS One. 2024 May 13;19(5):e0303184. doi: 10.1371/journal.pone.0303184 (PMC11090362; doi:10.1371/journal.pone.0303184)
Supplement: S2 File — (DOCX) [file pone.0303184.s002.docx]

**影响CSA现存会员续订意愿的影响因素**

1.您的性别是 [单选题] *

| ○男性 |
| --- |
| ○女性 |

2.您的年龄 [单选题] *

| ○18-29 |
| --- |
| ○30-44 |
| ○45-59 |
| ○60及以上 |

3.您的家庭成员有哪些？[矩阵单选题] *

|  | 1个 | 2个 | 3个 | 4个 | 5个 | 6个及以上 | 0个 |
| --- | --- | --- | --- | --- | --- | --- | --- |
| 2岁以下 | ○ | ○ | ○ | ○ | ○ | ○ | ○ |
| 2-6岁 | ○ | ○ | ○ | ○ | ○ | ○ | ○ |
| 7-17岁 | ○ | ○ | ○ | ○ | ○ | ○ | ○ |
| 18-29岁 | ○ | ○ | ○ | ○ | ○ | ○ | ○ |
| 30-44岁 | ○ | ○ | ○ | ○ | ○ | ○ | ○ |
| 45-59岁 | ○ | ○ | ○ | ○ | ○ | ○ | ○ |
| 60岁及以上 | ○ | ○ | ○ | ○ | ○ | ○ | ○ |

4.您的最高学历是什么？ [单选题] *

| ○研究生学历 |
| --- |
| ○本科学历 |
| ○大专或大专同等学历 |
| ○高中或高中同等学历 |
| ○高中以下学历 |

5.您的月收入是多少？ [单选题] *

| ○少于3000元 |
| --- |
| ○3000-5000元 |
| ○5000-8000元 |
| ○8000-15000元 |
| ○15000元以上 |

6.您目前从事的职业： [单选题] *

| ○公务人员 |
| --- |
| ○企业员工 |
| ○自己创业 |
| ○自由职业 |
| ○学生 |
| ○其他 |

7.我收到的CSA产品质量符合我的期望。 [单选题] *

| 很不满意 | ○1 | ○2 | ○3 | ○4 | ○5 | 很满意 |
| --- | --- | --- | --- | --- | --- | --- |

8.我认为产品价格符合我的预期。 [单选题] *

| 很不满意 | ○1 | ○2 | ○3 | ○4 | ○5 | 很满意 |
| --- | --- | --- | --- | --- | --- | --- |

9.CSA是我购买有机食品更好的选择。 [单选题] *

| 很不满意 | ○1 | ○2 | ○3 | ○4 | ○5 | 很满意 |
| --- | --- | --- | --- | --- | --- | --- |

10.CSA提供的农产品组合可以满足我的需求。 [单选题] *

| 很不满意 | ○1 | ○2 | ○3 | ○4 | ○5 | 很满意 |
| --- | --- | --- | --- | --- | --- | --- |

11.我可以订制“蔬菜箱”中农产品的种类和比例。 [单选题] *

| 很不满意 | ○1 | ○2 | ○3 | ○4 | ○5 | 很满意 |
| --- | --- | --- | --- | --- | --- | --- |

12.与农民直接交流，增强了我购买CSA服务的意愿。 [单选题] *

| 很不满意 | ○1 | ○2 | ○3 | ○4 | ○5 | 很满意 |
| --- | --- | --- | --- | --- | --- | --- |

13.CSA平台给会员之间提供了很多交流的机会。 [单选题] *

| 很不满意 | ○1 | ○2 | ○3 | ○4 | ○5 | 很满意 |
| --- | --- | --- | --- | --- | --- | --- |

14.在CSA中我能够遇见和我志同道合的朋友。 [单选题] *

| 很不满意 | ○1 | ○2 | ○3 | ○4 | ○5 | 很满意 |
| --- | --- | --- | --- | --- | --- | --- |

15.如果我有问题，我可以直接从我农场的工作人员那里得到解答。 [单选题] *

| 很不满意 | ○1 | ○2 | ○3 | ○4 | ○5 | 很满意 |
| --- | --- | --- | --- | --- | --- | --- |

16.我可以随时从CSA平台获取我农场的生产情况。 [单选题] *

| 很不满意 | ○1 | ○2 | ○3 | ○4 | ○5 | 很满意 |
| --- | --- | --- | --- | --- | --- | --- |

17.我很信任我的CSA农场。 [单选题] *

| 很不满意 | ○1 | ○2 | ○3 | ○4 | ○5 | 很满意 |
| --- | --- | --- | --- | --- | --- | --- |

18.我很信任农场的农民。 [单选题] *

| 很不满意 | ○1 | ○2 | ○3 | ○4 | ○5 | 很满意 |
| --- | --- | --- | --- | --- | --- | --- |

19.我相信CSA农场的产品符合CSA平台制定的产品质量规定。 [单选题] *

| 很不满意 | ○1 | ○2 | ○3 | ○4 | ○5 | 很满意 |
| --- | --- | --- | --- | --- | --- | --- |

20.我相信我的农场没有额外收取费用。 [单选题] *

| 很不满意 | ○1 | ○2 | ○3 | ○4 | ○5 | 很满意 |
| --- | --- | --- | --- | --- | --- | --- |

21.我相信我的农场在生产过程中坚持环保标准。 [单选题] *

| 很不满意 | ○1 | ○2 | ○3 | ○4 | ○5 | 很满意 |
| --- | --- | --- | --- | --- | --- | --- |

22.支持环保事业是我加入CSA的动力。 [单选题] *

| 很不满意 | ○1 | ○2 | ○3 | ○4 | ○5 | 很满意 |
| --- | --- | --- | --- | --- | --- | --- |

23.参与CSA在保护环境方面发挥着重要作用。 [单选题] *

| 很不满意 | ○1 | ○2 | ○3 | ○4 | ○5 | 很满意 |
| --- | --- | --- | --- | --- | --- | --- |

24.为了环境、健康和保护动物，我们应该减少肉类消费。 [单选题] *

| 很不满意 | ○1 | ○2 | ○3 | ○4 | ○5 | 很满意 |
| --- | --- | --- | --- | --- | --- | --- |

25.成为CSA成员是我们家庭采取的最重要的环保措施之一。 [单选题] *

| 很不满意 | ○1 | ○2 | ○3 | ○4 | ○5 | 很满意 |
| --- | --- | --- | --- | --- | --- | --- |

26.我可以步行到社区附近的取货点取货。 [单选题] *

| 很不满意 | ○1 | ○2 | ○3 | ○4 | ○5 | 很满意 |
| --- | --- | --- | --- | --- | --- | --- |

27.我可以乘坐公共交通到我附近的CSA农场取货。 [单选题] *

| 很不满意 | ○1 | ○2 | ○3 | ○4 | ○5 | 很满意 |
| --- | --- | --- | --- | --- | --- | --- |

28.我可以通过网上订购和送货到家服务得到我的产品。 [单选题] *

| 很不满意 | ○1 | ○2 | ○3 | ○4 | ○5 | 很满意 |
| --- | --- | --- | --- | --- | --- | --- |

29.我能在有需要的情况下及时的获得产品。 [单选题] *

| 很不满意 | ○1 | ○2 | ○3 | ○4 | ○5 | 很满意 |
| --- | --- | --- | --- | --- | --- | --- |

30.我认为订购CSA产品比在超市购买农产品更方便。 [单选题] *

| 很不满意 | ○1 | ○2 | ○3 | ○4 | ○5 | 很满意 |
| --- | --- | --- | --- | --- | --- | --- |

31.我将续订我所在CSA农场的会员。 [单选题] *

| 很不满意 | ○1 | ○2 | ○3 | ○4 | ○5 | 很满意 |
| --- | --- | --- | --- | --- | --- | --- |

32.我会推荐我的朋友和亲戚加入CSA。 [单选题] *

| 很不满意 | ○1 | ○2 | ○3 | ○4 | ○5 | 很满意 |
| --- | --- | --- | --- | --- | --- | --- |

33.我喜欢CSA平台提供的服务。 [单选题] *

| 很不满意 | ○1 | ○2 | ○3 | ○4 | ○5 | 很满意 |
| --- | --- | --- | --- | --- | --- | --- |

34.我会将我在CSA农场的体验分享到社交平台。 [单选题] *

| 很不满意 | ○1 | ○2 | ○3 | ○4 | ○5 | 很满意 |
| --- | --- | --- | --- | --- | --- | --- |
