# Supplementary material for: Investigating consumers’ experiences with community supported agriculture: Convergent parallel design methods
Source: PLoS One. 2024 May 13;19(5):e0303184. doi: 10.1371/journal.pone.0303184 (PMC11090362; doi:10.1371/journal.pone.0303184)
Supplement: S3 File — S1 Table. This is the S1 Table measurement. S2 Fig. This is the S2 Fig P-P, Q-Q. PS: Product Satisfaction, I: Interaction, T: Trust, EM: Environmental Motivation, C: Convenience, R: Retention, Intention. S3 Table. This is the S3 Table The demographic information of research samples in phase one (N = 250). S4 Table. This is the S4 Table Communalities. S5 Table. This is the S5 Table Total Variance Explained. S6 Table. This is the S6 Table Stepwise multiple linear regression. (PDF) [file pone.0303184.s003.pdf]

Supporting information

S1 Table. This is the S1 Table measurement.

Construct	Item	Resource of measurement
Product Satisfaction	PS1Q7. The quality of my CSA produce meets my expectations.<br>PS2Q8. The price of the harvest share is appropriate.<br>PS3Q9. It is a better selection of organic food.<br>PS4Q10. The produce mix offered by CSA met my needs.<br>PS5Q11.I can customize the types and proportions of produce in the "Vegetable Box".	Christine Hvitsand 2016<br>Ryan E. Galta, et al.,2019
Interaction	I1Q12. Interacting directly with farmers has strengthened my willingness to purchase CSA services.<br>I2Q13. In my CSA there are sufficient opportunities for member participation.<br>I3Q14. To meet like-minded people in farm is important to me.<br>I4Q15. If I have questions, I can get answers directly from the staff on my farm.<br>I5Q16. It is important to me to have the possibility to visit the CSA to personally get an idea of the production of my food.	Felix Zoll, et al., 2022<br>Antoinette Pole, et al.,2013
Trust	T1Q17. I generally trust in my CSA.<br>T2Q18. I generally trust in the farmer(s) of my CSA.<br>T3Q19. I believe that the products of CSA farms meet the product quality regulations set by the CSA platform.<br>T4Q20. I trust that my CSA does not overcharge for their products.<br>T5Q21. I trust my farm to uphold environmental standards during production.	Felix Zoll, et al., 2022
Environmental Motivation	EM1Q22. Supporting environmentally friendly practices is my motivation for joining CSA.<br>EM2Q23. Participation in my CSA plays an important role in improving and protecting the environment.<br>EM3Q24. We should reduce the consumption of meat in favor of environment, health and animals.	Christine Hvitsand 2016<br>Danielle del Castillo Shelton, B.S.2012

	EM4Q25. To be a CSA member is one of the most important environmental measures our household does.	
Convenience	C1Q26. I can walk to a community pickup location to pick-up my order.<br>C2Q27. I can pick-up produce on the farm with transportation.<br>C3Q28. I can get my products through online order and delivery service.<br>C4Q29. I was able to get my product just in time when I wanted it.<br>C5Q30. I find it easier to order at CSA than to buy produce at the supermarket.	Junhong Chen, et al.,2019<br>Ruoding Shi, et al.,2015<br>Baudouin Q.2010
Retention Intention	R1Q31. I will continue to renew my CSA membership.<br>R2Q32. I would recommend to people who are important to me to join my CSA.<br>R3Q33. I am satisfied with the services provided by the CSA.<br>R4Q34. I will share my experience in CSA on social platforms.	Baudouin Q.2010

The scale ranges from 1 to 5, 1 = Strongly Disagree, 2 = Disagree, 3 = Neutral, 4 = Agree, 5 = Strongly Agree

S2 Figure. This is the S2 Figure P-P,   Q-Q.

PS: Product Satisfaction, *I*: Interaction, *T*: Trust, *EM*: Environmental Motivation, *C*: Convenience, *R*: Retention Intention

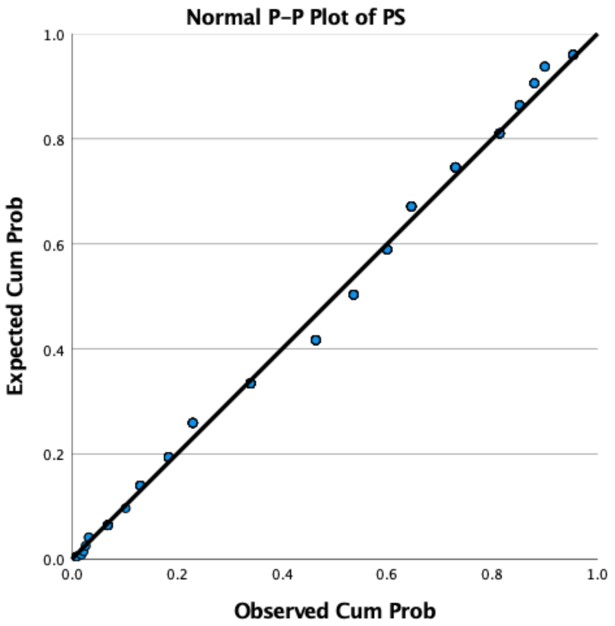

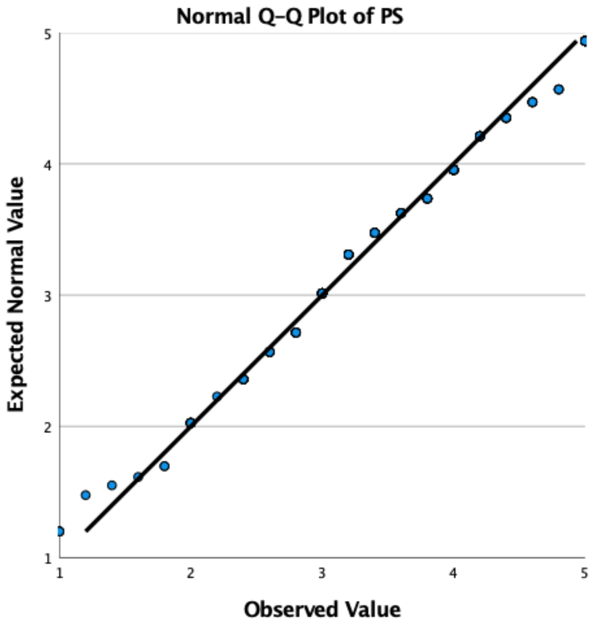

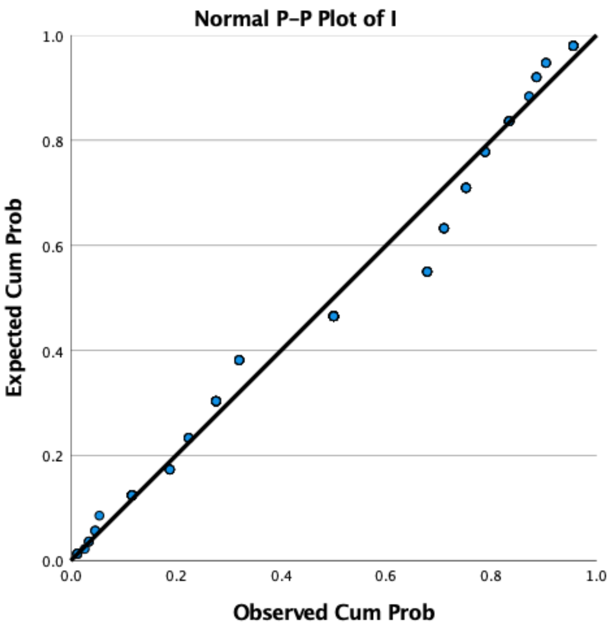
Normal P-P Plot of I
Expected Cum Prob
Observed Cum Prob
0.0
0.2
0.4
0.6
0.8
1.0

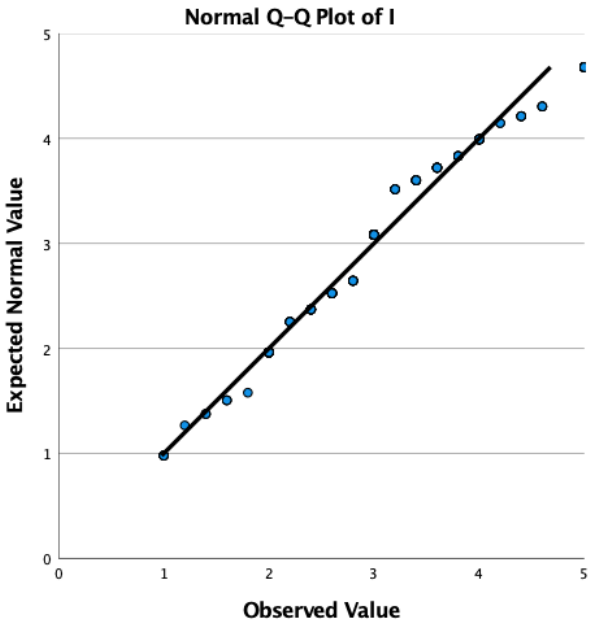
Normal Q-Q Plot of I
Expected Normal Value
Observed Value
0
1
2
3
4
5

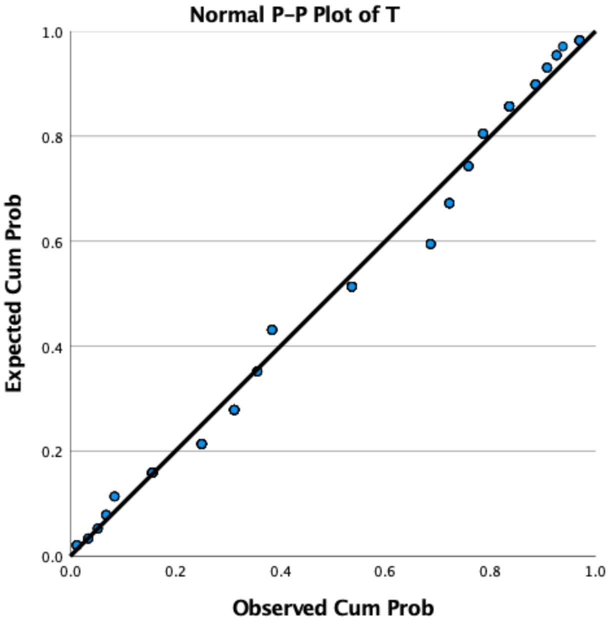
Normal P-P Plot of T
Expected Cum Prob
Observed Cum Prob
0.0
0.2
0.4
0.6
0.8
1.0

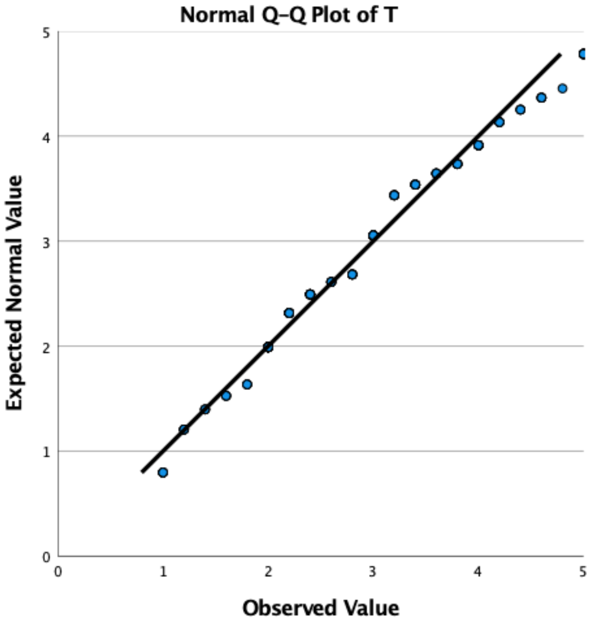
Normal Q-Q Plot of T
Expected Normal Value
Observed Value
0
1
2
3
4
5

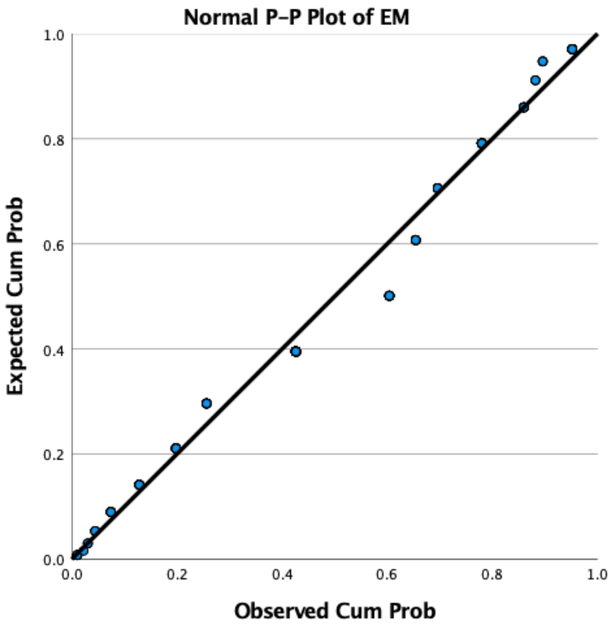

Normal P-P Plot of EM
Expected Cum Prob
Observed Cum Prob
0.0
0.2
0.4
0.6
0.8
1.0

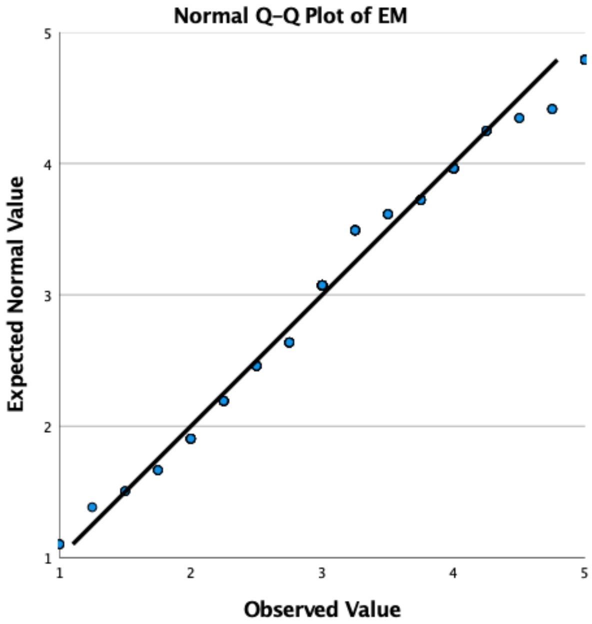

Normal Q-Q Plot of EM
Expected Normal Value
Observed Value
1
2
3
4
5

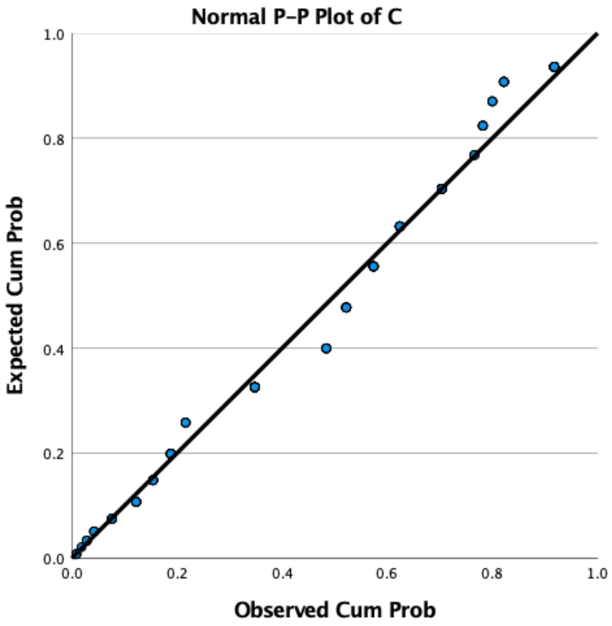
Normal P-P Plot of C
Expected Cum Prob
Observed Cum Prob
0.0
0.2
0.4
0.6
0.8
1.0

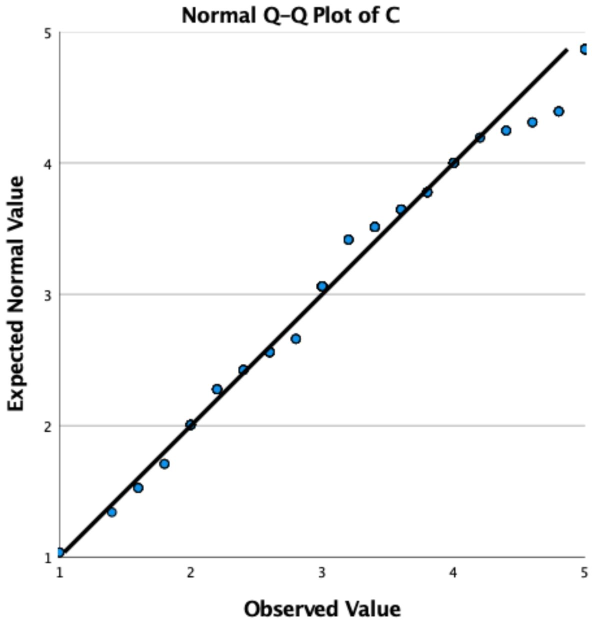
Normal Q-Q Plot of C
Expected Normal Value
Observed Value
1
2
3
4
5

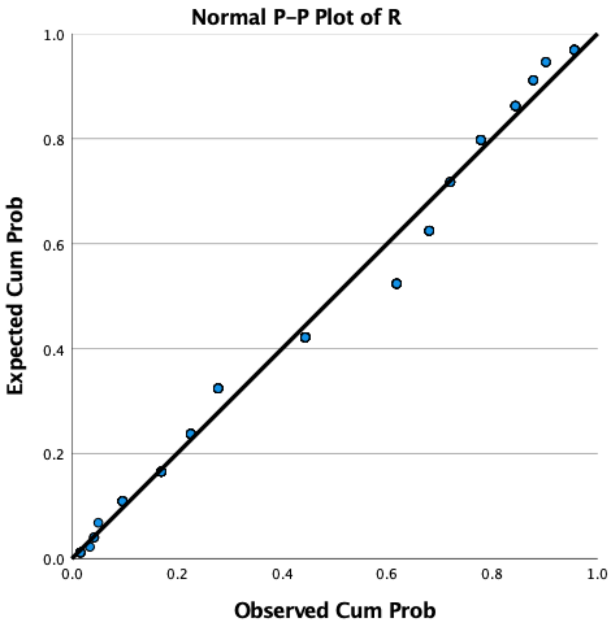
Normal P-P Plot of R
Expected Cum Prob
Observed Cum Prob
0.0
0.2
0.4
0.6
0.8
1.0

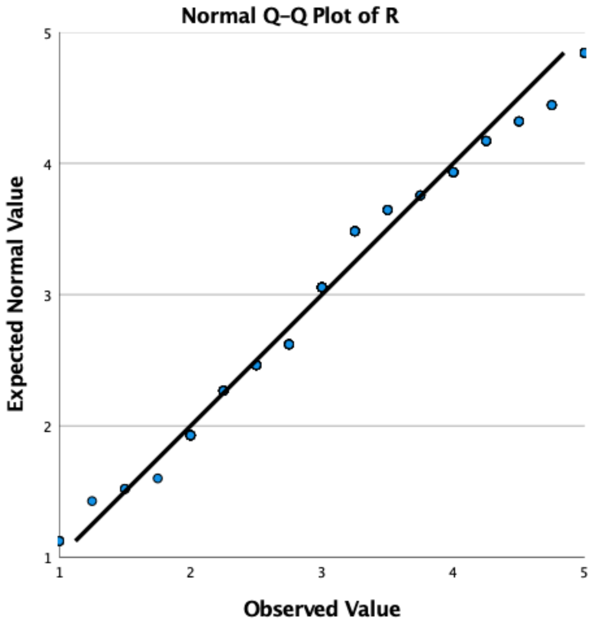
Normal Q-Q Plot of R
Expected Normal Value
Observed Value
1
2
3
4
5

S3 Table. This is the S3 Table The demographic information of research samples in phase one (N=250).

Variables		N (%)	Variables		N (%)
Gender			Education		
	Male	119 (47.6)		Graduate	14 (5.6)
	Female	131 (52.4)		Undergraduate	103 (41.2)
Age				Junior College	103 (41.2)
	18-29	93 (37.2)		High school	37 (14.8)
	30-44	100 (40)		Less than high school	5 (2)
	45-59	47 (18.8)	Income (monthly)		
	Age 60 and above	10 (4)		3000-5000	42 (16.8)
				5000-8000	159 (63.6)
				8000-15000	36 (14.4)
				Above 15,000	13 (5.2)

S4 Table. This is the S4 Table Communalities.

Communalities		
	Initial	Extraction
PS1Q7	1.000	.744
PS2Q8	1.000	.806
PS3Q9	1.000	.793
PS4Q10	1.000	.765
PS5Q11	1.000	.746
I1Q12	1.000	.817
I2Q13	1.000	.870
I3Q14	1.000	.871
I4Q15	1.000	.914
I5Q16	1.000	.853
T1Q17	1.000	.874
T2Q18	1.000	.819
T3Q19	1.000	.821
T4Q20	1.000	.849
T5Q21	1.000	.846
EM1Q22	1.000	.825
EM2Q23	1.000	.891
EM3Q24	1.000	.835
EM4Q25	1.000	.767
C1Q26	1.000	.906
C2Q27	1.000	.826
C3Q28	1.000	.899
C4Q29	1.000	.892
C5Q30	1.000	.851
R1Q31	1.000	.860
R2Q32	1.000	.776
R3Q33	1.000	.810
R4Q34	1.000	.868

Extraction Method: Principal Component Analysis.

S5 Table. This is the S5 Table Total Variance Explained.

Total Variance Explained

Component	Initial Eigenvalues			Extraction Sums of Squared Loadings			Rotation Sums of Squared Loadings		
	Total	% of Variance	Cumulative %	Total	% of Variance	Cumulative %	Total	% of Variance	Cumulative %
1	15.008	53.600	53.600	15.008	53.600	53.600	4.513	16.116	16.116
2	2.517	8.990	62.589	2.517	8.990	62.589	4.389	15.676	31.792
3	1.980	7.072	69.662	1.980	7.072	69.662	4.180	14.930	46.722
4	1.462	5.220	74.882	1.462	5.220	74.882	4.052	14.470	61.192
5	1.249	4.460	79.342	1.249	4.460	79.342	3.223	11.510	72.702
6	1.178	4.205	83.548	1.178	4.205	83.548	3.037	10.846	83.548
7	.516	1.843	85.391						
8	.429	1.531	86.921						
9	.338	1.207	88.129						
10	.332	1.185	89.314						
11	.300	1.070	90.384						
12	.263	.940	91.323						
13	.240	.858	92.182						
14	.239	.854	93.035						
15	.224	.798	93.834						
16	.210	.751	94.585						
17	.194	.691	95.277						
18	.177	.631	95.907						
19	.152	.543	96.450						
20	.147	.525	96.975						
21	.137	.491	97.466						
22	.127	.454	97.920						
23	.118	.422	98.341						
24	.117	.418	98.759						
25	.103	.369	99.128						
26	.095	.338	99.467						
27	.080	.284	99.751						
28	.070	.249	100.000						

Extraction Method: Principal Component Analysis.

S6 Table. This is the S6 Table Stepwise multiple linear regression.

Variables Entered/Removed[a]

Model	Variables Entered	Variables Removed	Method
1	I	.	Stepwise (Criteria: Probability-of-F-to-enter <= .050, Probability-of-F-to-remove >= .100).
2	C	.	Stepwise (Criteria: Probability-of-F-to-enter <= .050, Probability-of-F-to-remove >= .100).
3	T	.	Stepwise (Criteria: Probability-of-F-to-enter <= .050, Probability-of-F-to-remove >= .100).
4	PS	.	Stepwise (Criteria: Probability-of-F-to-enter <= .050, Probability-of-F-to-remove >= .100).
5	EM	.	Stepwise (Criteria: Probability-of-F-to-enter <= .050, Probability-of-F-to-remove >= .100).

a. Dependent Variable: R

Model Summary[f]

Model	R	R Square	Adjusted R Square	Std. Error of the Estimate	Change Statistics					Durbin-Watson
					R Square Change	F Change	df1	df2	Sig. F Change	
1	.625[a]	.391	.389	.75837	.391	159.358	1	248	.000	
2	.721[b]	.520	.516	.67482	.129	66.213	1	247	.000	
3	.741[c]	.549	.543	.65573	.029	15.594	1	246	.000	
4	.752[d]	.566	.559	.64449	.017	9.653	1	245	.002	
5	.759[e]	.576	.567	.63831	.010	5.770	1	244	.017	1.819

a. Predictors: (Constant), I

b. Predictors: (Constant), I, C

c. Predictors: (Constant), I, C, T

d. Predictors: (Constant), I, C, T, PS

e. Predictors: (Constant), I, C, T, PS, EM

f. Dependent Variable: R

Coefficients[a]

Model		Unstandardized Coefficients		Standardized Coefficients			Correlations			Collinearity Statistics	
		B	Std. Error	Beta	t	Sig.	Zero-order	Partial	Part	Tolerance	VIF
1	(Constant)	1.198	.165		7.259	.000					
	I	.647	.051	.625	12.624	.000	.625	.625	.625	1.000	1.000
2	(Constant)	.460	.173		2.664	.008					
	I	.451	.052	.436	8.756	.000	.625	.487	.386	.783	1.278
	C	.388	.048	.406	8.137	.000	.609	.460	.359	.783	1.278
3	(Constant)	.354	.170		2.083	.038					
	I	.333	.058	.322	5.714	.000	.625	.342	.245	.577	1.733
	C	.327	.049	.341	6.687	.000	.609	.392	.286	.704	1.421
	T	.230	.058	.229	3.949	.000	.606	.244	.169	.544	1.837
4	(Constant)	.180	.176		1.024	.307					
	I	.259	.062	.251	4.181	.000	.625	.258	.176	.492	2.030
	C	.263	.052	.275	5.025	.000	.609	.306	.212	.594	1.683
	T	.222	.057	.222	3.880	.000	.606	.241	.163	.543	1.840
	PS	.190	.061	.180	3.107	.002	.595	.195	.131	.528	1.894
5	(Constant)	.102	.177		.577	.564					
	I	.211	.065	.204	3.260	.001	.625	.204	.136	.444	2.250
	C	.224	.054	.234	4.135	.000	.609	.256	.172	.542	1.845
	T	.193	.058	.193	3.329	.001	.606	.208	.139	.520	1.924
	PS	.176	.061	.167	2.895	.004	.595	.182	.121	.523	1.912
	EM	.152	.063	.146	2.402	.017	.613	.152	.100	.472	2.120

a. Dependent Variable: R
